# Supplementary material for: A minimal biochemical route towards de novo formation of synthetic phospholipid membranes
Source: Nat Commun. 2019 Jan 17;10:300. doi: 10.1038/s41467-018-08174-x (PMC6336818; doi:10.1038/s41467-018-08174-x)
Supplement: Supplementary file 1 — Supplementary Information [file 41467_2018_8174_MOESM1_ESM.pdf]

Supplementary Information

A Minimal Biochemical Route towards *de novo* Formation of Synthetic Phospholipid Membranes

Bhattacharya *et al.*

Supplementary Figures

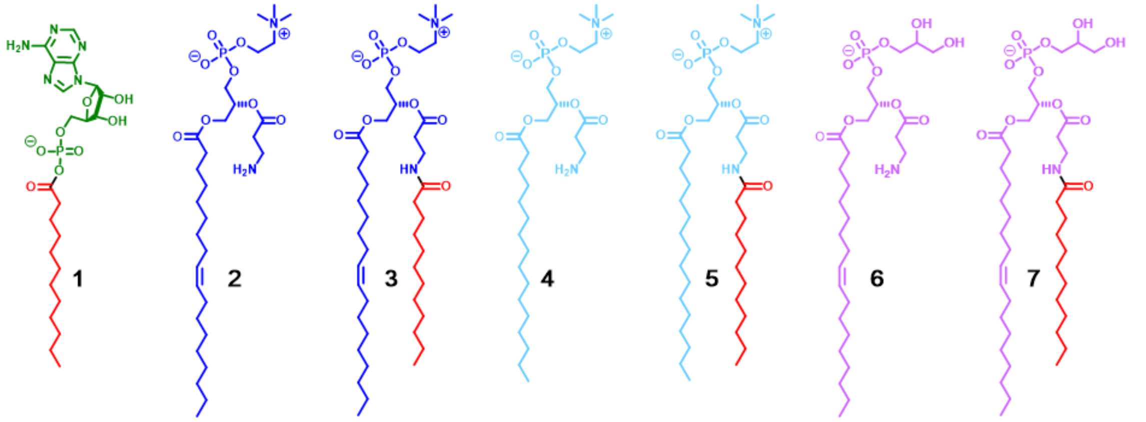

Supplementary Figure 1. Chemical structures of all the lipids used in this study. The names of the lipids according to their numbering are as follows: **1**. Dodecanoyl-AMP **2**. 1-oleoyl-2-(β-Ala)-*sn*-glycero-3-phosphocholine **3**. 1-oleoyl-2-[β-Ala-(dodecanoyl)]-*sn*-glycero-3-phosphocholine **4**. 1-palmitoyl-2-(β-Ala)-*sn*-glycero-3-phosphocholine **5**. 1-palmitoyl-2-[β-Ala-(dodecanoyl)]-*sn*-glycero-3-phosphocholine **6**. 1-oleoyl-2-(β-Ala)-*sn*-glycero-3-phosphoglycerol **7**. 1-oleoyl-2-[β-Ala-(dodecanoyl)]-*sn*-glycero-3-phosphoglycerol.

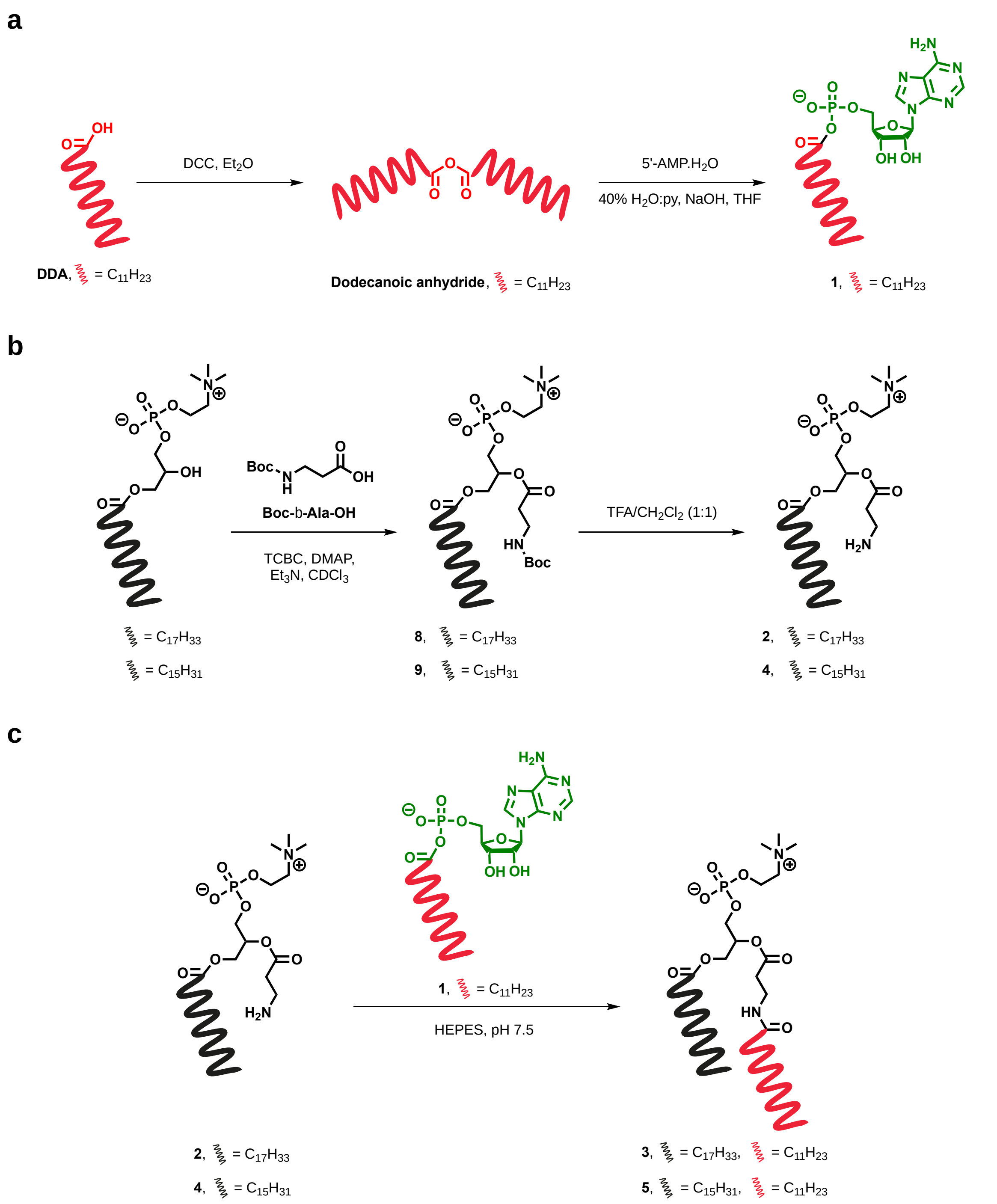

Supplementary Figure 2. Formation of phospholipid membranes based on adenylate chemistry. (**a**) Synthesis of dodecanoyl-AMP (**1**). (**b**) Synthesis of amine-functionalized lysolipids **2** and **4**. (**c**) *De novo* synthesis of phospholipids (**3** or **5**) by chemoselective reaction of amine-functionalized lysolipids (**2** or **4**) and dodecanoyl-AMP (**1**).

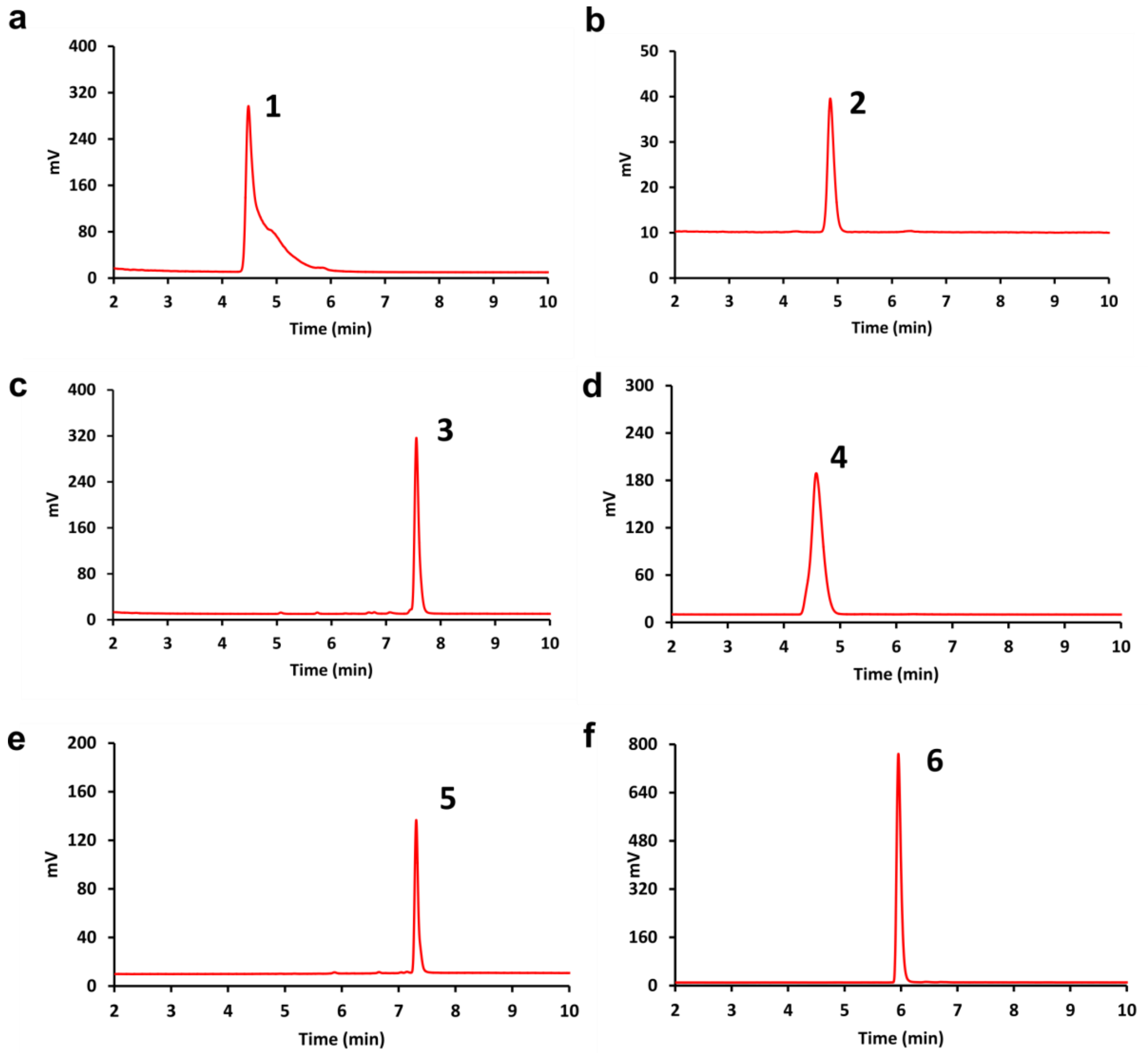

Supplementary Figure 3. HPLC/ELSD traces of the synthetic lipids used in this study. (**a**)Dodecanoyl-AMP **1**. (**b**) Lysolipid **2**. (**c**) Phospholipid **3**. (**d**) Lysolipid **4**. (**e**) Phospholipid **5** (**f**) Lysolipid **6**. Retention times for all the species were verified by mass spectrometry.

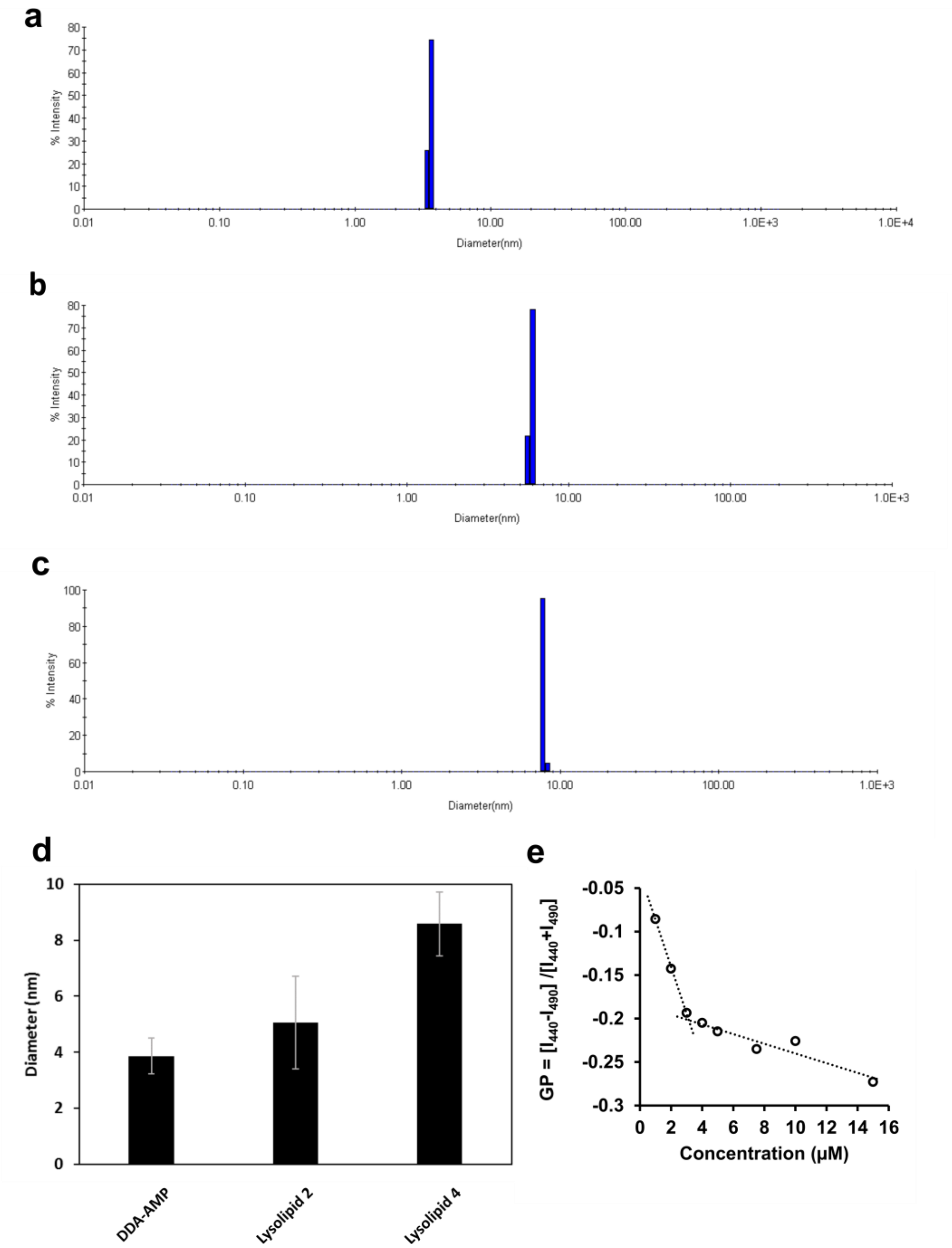

Supplementary Figure 4. Self-assembly properties of DDA-AMP and lysolipids. Dynamic Light Scattering (DLS) plots corresponding to a 1 mM solution of dodecanoyl-AMP **1** (**a**), lysolipid **2** (**b**), and lysolipid **4** (**c**). (**d**) Estimation of micelle sizes of dodecanoyl-AMP **1**, lysolipid **2** and lysolipid **4** each at 1 mM concentration using dynamic light scattering. The error bars represent standard deviation (n = 3). (**e**) Critical micelle concentration (cmc) of lysolipid **2** was determined according to a previously published method[1] by plotting the values of generalized polarization (GP = $[I_{440}-I_{490}]/[I_{440}+I_{490}]$) of the chemical environment-sensitive dye Laurdan at various concentrations of the lysolipid. A sharp change in the GP values is observed at about 3 µM. This suggests that the cmc value of the lysolipid is at this concentration.

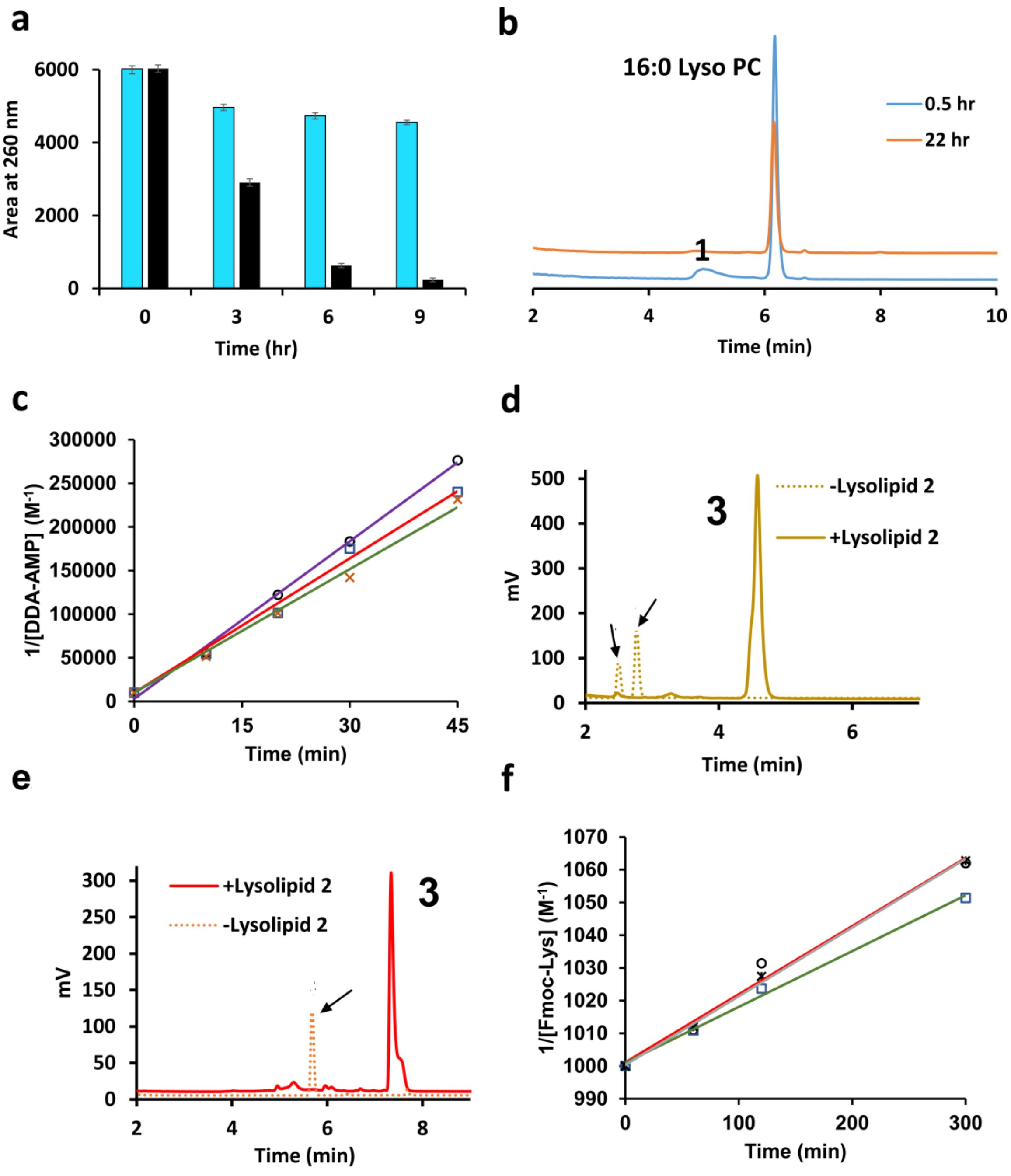

Supplementary Figure 5. Stability and reactivity studies of dodecanoyl-AMP (**1**). (**a**) Stability of **1** over time in the absence or presence (10 mM) of Mg^{2+}. The cyan bars represent integrated HPLC peak areas (260 nm) for the **1** in the absence of Mg^{2+}, whereas the black bars represent integrated HPLC peak areas (260 nm) for **1** in the presence of 10 mM Mg^{2+}. The error bars represent standard deviation (n = 3). (**b**) Experiment showing the unreactive nature of 1-palmitoyl-2-hydroxy-*sn*-glycero-3-phosphocholine (Lyso $C_{16:0}$ PC-OH) towards **1**. HPLC/ELSD traces monitoring the progress of a reaction mixture containing Lyso $C_{16:0}$ PC-OH (1 mM) and **1** (1 mM) in the presence of 9 mM $MgCl_2$ in

50 mM HEPES pH 7.5 buffer. Even after 22 h, no phospholipid formation was detectable. The retention times of all peaks were verified by mass spectrometry. (**c**) Second order kinetic plots corresponding to the reaction between 0.1 mM **1** and lysolipid **2** at 37 ^{o}C. The rate constant was calculated from three independent trials. (**d**) HPLC-ELSD-MS chromatograms showing the selectivity of the reaction between 1 mM of each of **1** and lysolipid **2** in presence of 50 mM lysine and forming phospholipid **3** with high selectivity. The dotted chromatogram corresponds to a control reaction where the lysolipid was omitted. The arrows indicate the dodecanoyl amides formed by the reaction with the α-NH_2 and ε-NH_2 groups of lysine. (**e**) HPLC-ELSD-MS chromatograms showing the selectivity of the reaction between 1 mM of each of adenylate **1** and lysolipid **2** in presence of 100 mM Tris buffer (pH 8.0). The dotted chromatogram corresponds to a control reaction where the lysolipid was omitted. The arrow indicates the dodecanoyl amide formed by the reaction with the -NH_2 group of Tris. (**f**) Second order kinetic plots corresponding to the reaction between 1.0 mM **1** and Fmoc-Lys-OH at 37 ^{o}C. The rate constant was calculated from three independent trials.

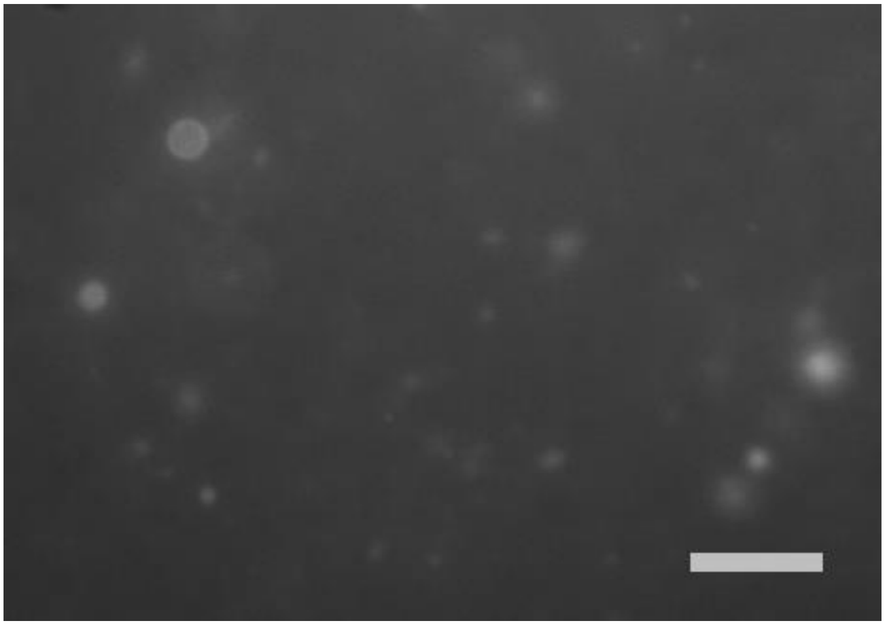

Supplementary Figure 6. *In situ* formation of phospholipid **3** vesicles. The phospholipid **3** is generated by the reaction of FAA **1** and lysolipid **2**. Membranes were stained using Texas Red® DHPE dye (0.1 mol%). Scale bar represents 10 µm.

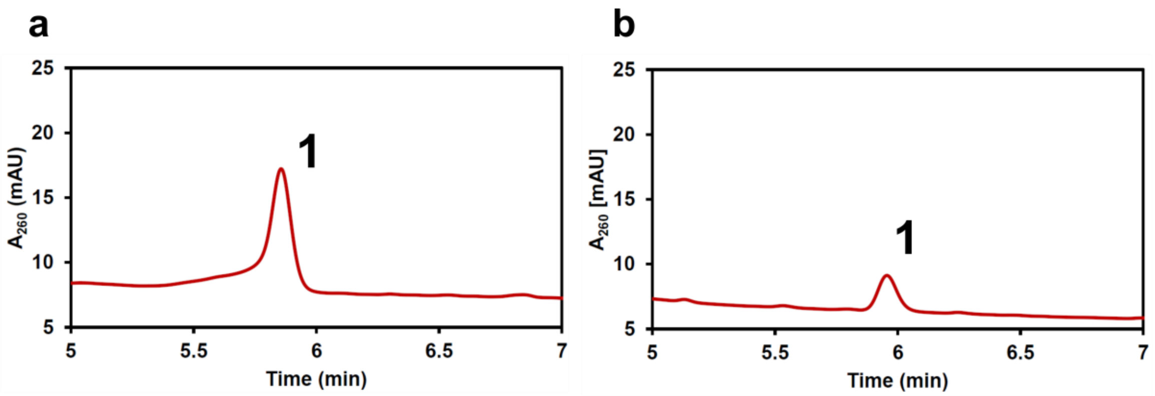

Supplementary Figure 7. Dodecanoyl-AMP intermediate generated by FadD10. (**a**) HPLC chromatogram (260 nm) showing the formation of the dodecanoyl-AMP intermediate **1** in a reaction mixture containing sodium dodecanoate (0.2 mM), $MgCl_2$ (8 mM), ATP (2 mM) and FadD10 (10 µM) incubated at 37 °C in 50 mM HEPES buffer (pH 7.5) for 1 h. The reaction mixture was directly injected without further purification. (**b**) HPLC chromatogram (260 nm) showing dodecanoyl-AMP intermediate **1** obtained by spin filtration (3 kDa MWCO) of reaction mixture. The indicated peaks were verified by mass spectrometry.

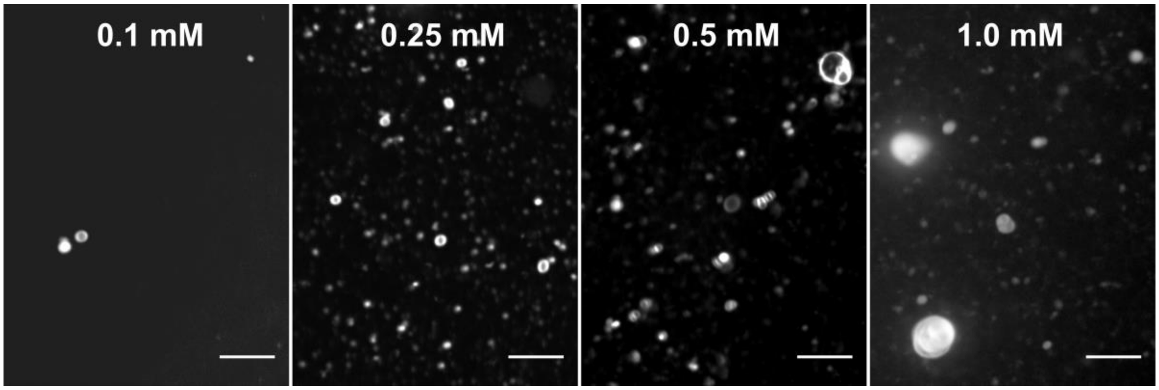

Supplementary Figure 8. FadD10 assisted phospholipid membrane formation. Formation of phospholipid **3** membrane formation in 100 mM HEPES (pH 8.0) at 37 °C from various concentrations of amphiphilic precursors (each of lysolipid **2** and sodium dodecanoate) as indicated assisted by FadD10. The membranes are stained with Texas Red-DHPE. All scale bars represent 10 µm.

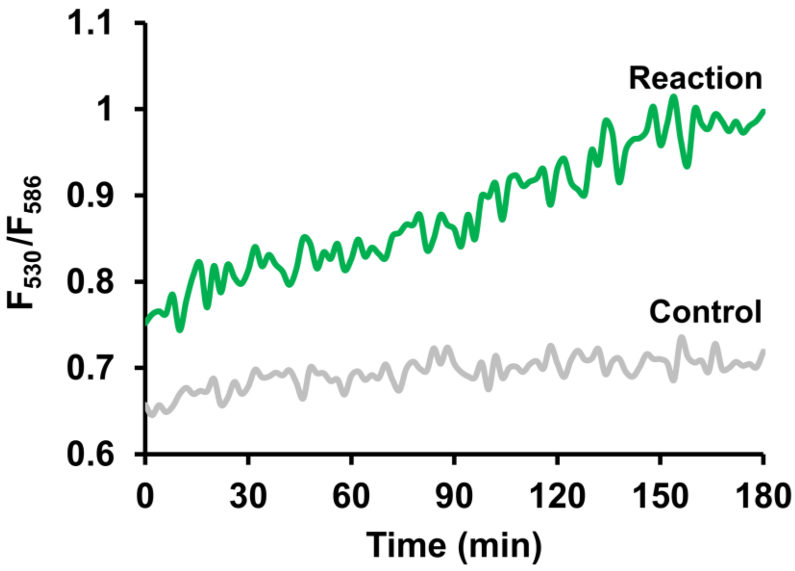

Supplementary Figure 9. FRET assay for *de novo* phospholipid membrane formation. Assaying *de novo* membrane formation using FRET between 0.2 mol% of each of NBD-DHPE (donor; λ_{ex}: 430 nm, λ_{em}: 530 nm) and Rhodamine-DHPE (acceptor; λ_{ex}: 560 nm, λ_{em}: 586 nm). The fluorophores were excited at 430 nm (bandwidth: 7.5 nm) and emission acquired at 530 nm (bandwidth: 7.5 nm) and 586 nm (bandwidth: 7.5 nm). The *green* trace shows a gradual increase in the F_{530}/F_{586} corresponding to *de novo* membrane growth arising from phospholipid synthesis (Reaction). The *grey* trace corresponds to a control condition where ATP is substituted with GTP and no phospholipid synthesis took place (Control).

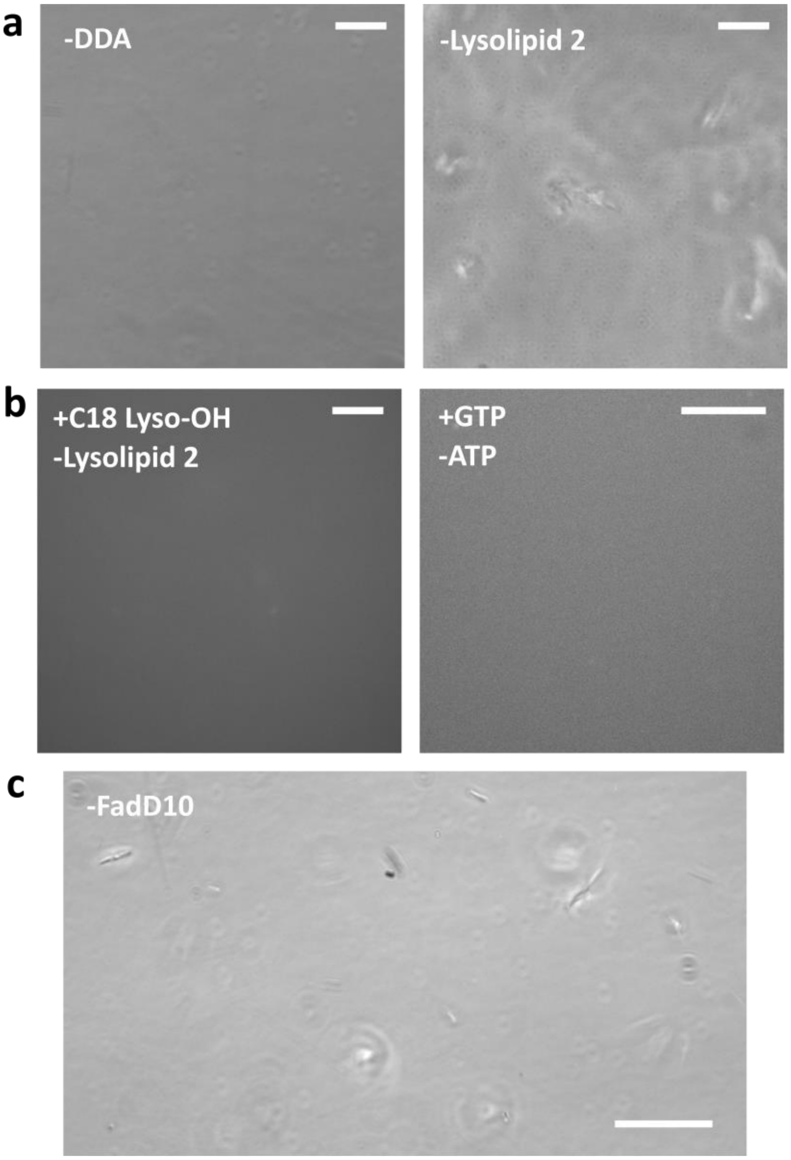

Supplementary Figure 10. Control experiments for *de novo* phospholipid formation. (**a**) Conditions where one of the precursors were omitted: *left* image (bright field) – omission of dodecanoic acid resulted in a clear solution; *right* image (bright field) – omission of lysolipid **2** resulted in aggregate formation, possibly due to precipitation of fatty acid. Scale bars denote 10 µm. (**b**) Conditions where a precursor was substituted by a closely related molecule: *left* image (Texas Red channel) – lysolipid **2** replaced by unreactive Lyso $C_{18:1}$ PC-OH resulted in a clear solution; *right* image (Texas Red channel) – replacement of ATP with GTP resulted in a clear solution as well. In these experiments, 0.1 mol% of the membrane staining dye Texas Red® DHPE was added to visualize if any membranous structures were formed. Scale bars denote 10 µm. (**c**) When FadD10 was omitted, a mostly clear solution with few small aggregates was obtained. Scale bar denotes 20 µm. The control conditions had the same compositions and temperature (37 ^{o}C) throughout as the reactive mixture except the changes and were checked up to 24 h and no vesicles were noticed in each case.

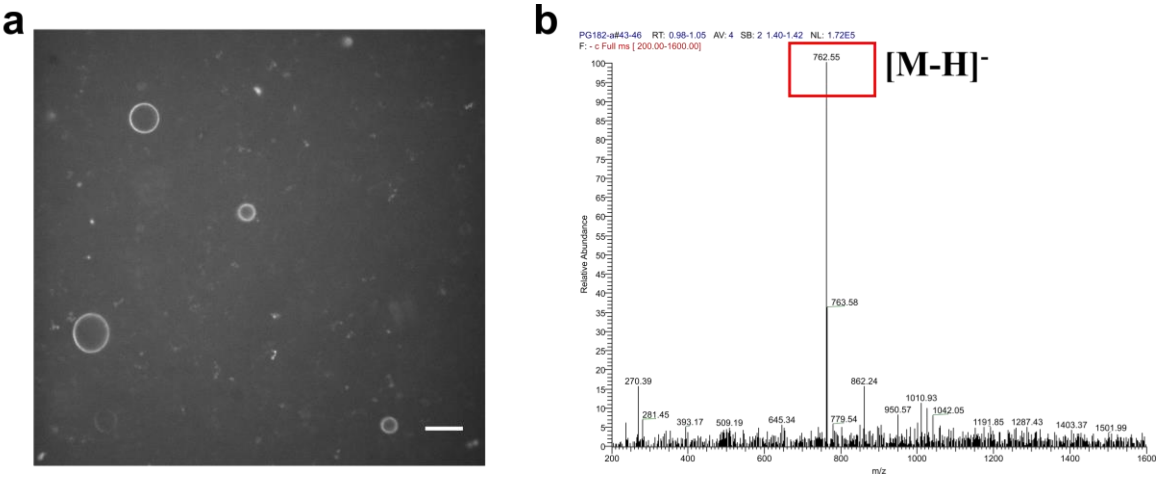

Supplementary Figure 11. Formation of phosphatidylglycerol lipid. (**a**) FadD10 assisted formation of the anionic phosphoglycerol head group bearing phospholipid **7** in 100 mM HEPES (pH 8.0) at 37 ^{o}C. The membranes are stained with 0.04 mol% Texas Red-DHPE. Scale bar: 10 µm. (**b**) Detection of FadD10-assisted *in situ* formed phospholipid **7** by ESI-MS (negative ion mode). The peak with *m/z* = 762.55 corresponds to the species [M-H]$^-$.

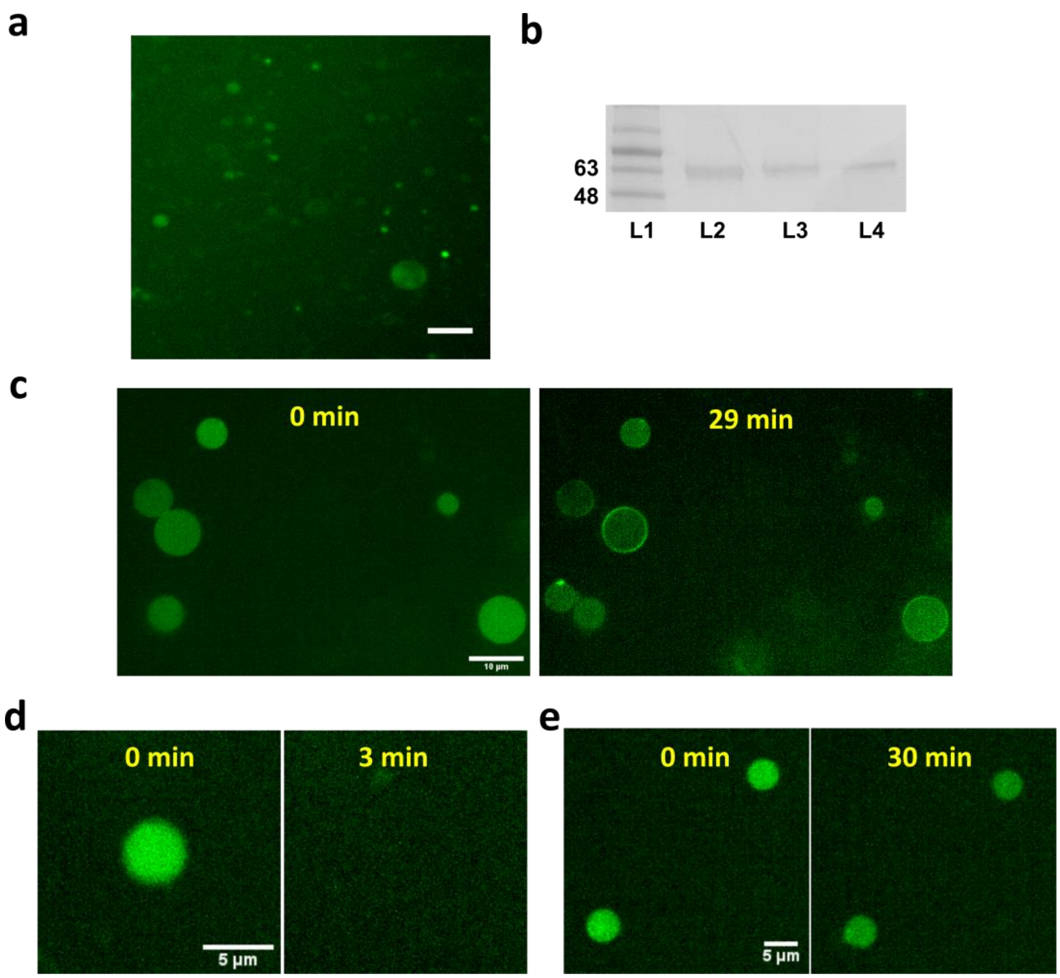

Supplementary Figure 12. Membrane association of FadD10. (**a**) *De novo* formation of vesicles in presence of Alexa Fluor 488-FadD10 followed by proteinase K treatment and spin filtering (10 kDa MWCO). Scale bar: 5 µm. (**b**) SDS-PAGE analysis of the encapsulation of FadD10 within *de novo* formed phospholipid **3** vesicles. Lane **L1**: molecular-weight size marker (63 and 48 kDa bands indicated); Lane **L2**: Crude reaction mixture; Lane **L3**: Vesicle fraction after spin-filter purification; Lane **L4**: Concentrated wash fraction after spin-filtration. (**c**) Localization of Alexa Fluor® 488 FadD10 encapsulated in DOPC GUVs following slow addition of membrane forming precursors (dodecanoic acid, lysolipid **4**, Mg^{2+} and ATP). Scale bar: 10 µm. (**d**) DOPC GUVs encapsulating Alexa Fluor® 488 FadD10 showed rapid leakage of the protein when lysolipid **4**, Mg^{2+} and ATP are added only. Scale bar: 5 µm. (**e**) DOPC GUVs encapsulating Alexa Fluor® 488 FadD10 imaged at interval of 30 min when no precursors are added. Scale bar: 5 µm.

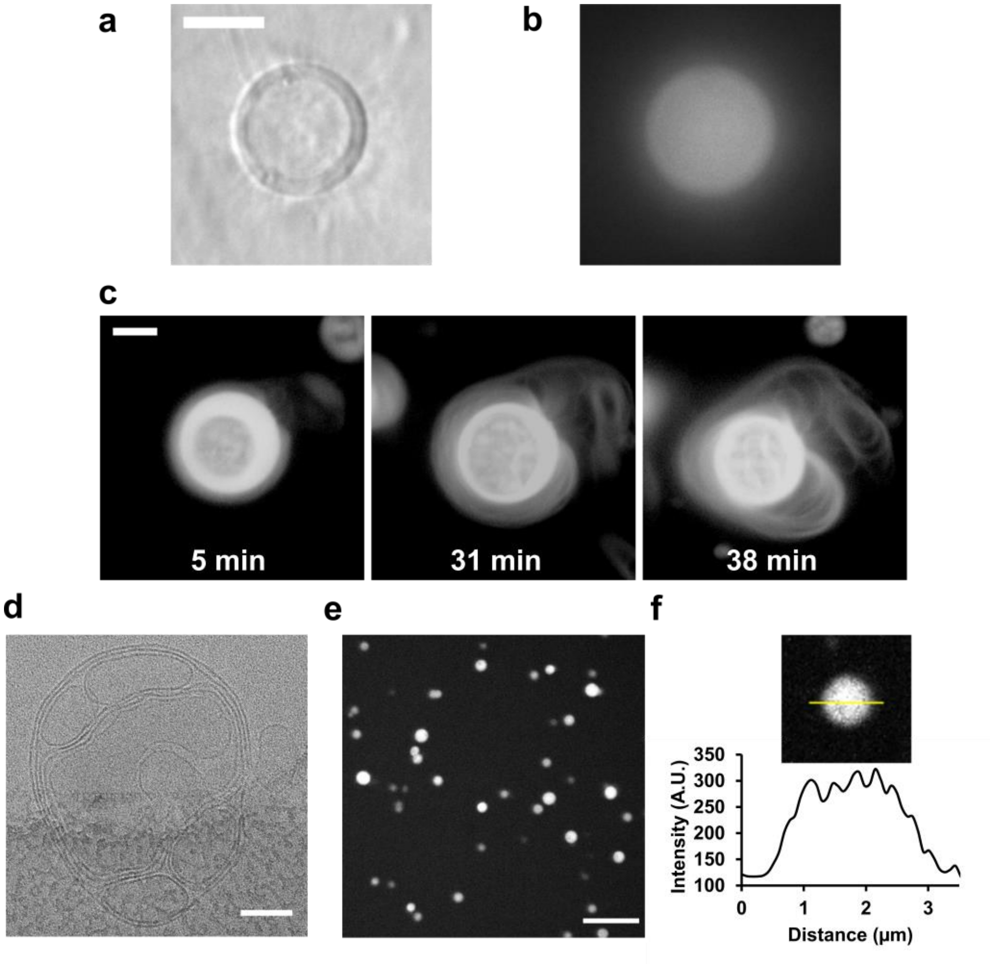

Supplementary Figure 13. Characterization of the vesicles used in the microfluidic experiments. (**a**) Bright field and (**b**) Fluorescence microscopy of a vesicle used for microfluidics experiments (Scale bar: 10 µm). (**c**) Fluorescence microscopy images (with logarithmic scaling) monitoring the osmotic swelling of phospholipid **3** vesicles encapsulating FadD10, revealing their internal membrane structure. (Scale bar: 10 µm). (**d**) Cryo-electron micrograph of a vesicle used in the microfluidics experiments. Scale bar denotes 100 nm. (**e**) Encapsulation of HPTS dye by the vesicles when the dye is added to the reaction mixture. Scale bar denotes 10 µm. (**f**) When 0.1 mM TNP-ATP is added the reaction mixture during phospholipid **3** vesicle formation, significantly high fluorescence signal could be detected in the vesicles over the background, suggesting that FadD10 has been encapsulated in a functional (ATP-binding) form. A representative vesicle is shown with the corresponding plot of fluorescence intensity profile along a straight line passing through the center.

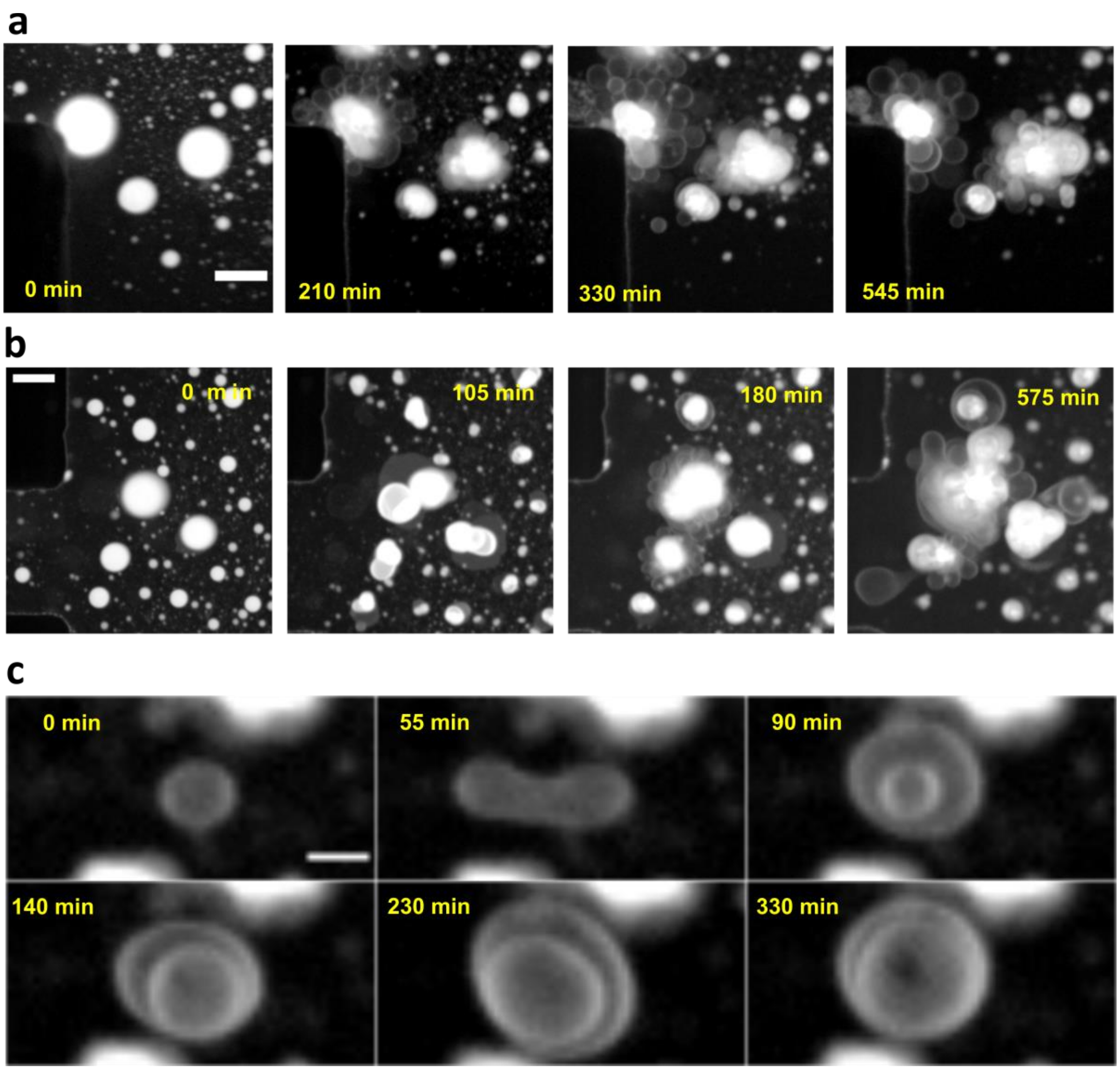

Supplementary Figure 14. Growth of vesicles on a microfluidic device. (**a-c**) The images are shown in Texas Red® channel (membrane) and scaled logarithmically for better visualization of fine structures. In **a** and **b**, the scale bars represent 20 µm. In **c**, the scale bar represents 5 µm.

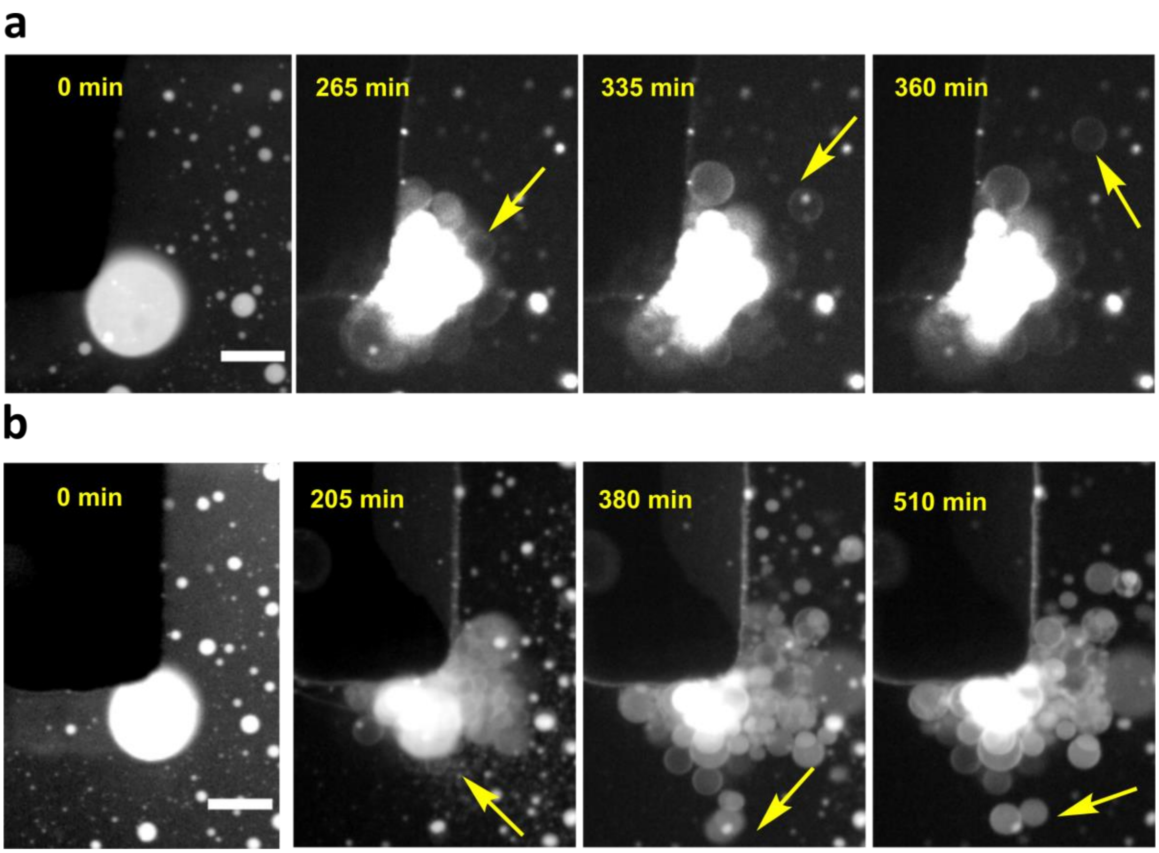

Supplementary Figure 15. Division of vesicles on a microfluidic device. (**a, b**) The images are shown in Alexa Fluor® 488 channel (labeled enzyme) and scaled logarithmically. Formation of representative daughter vesicles is indicated by yellow arrows. Scale bars represent 20 µm.

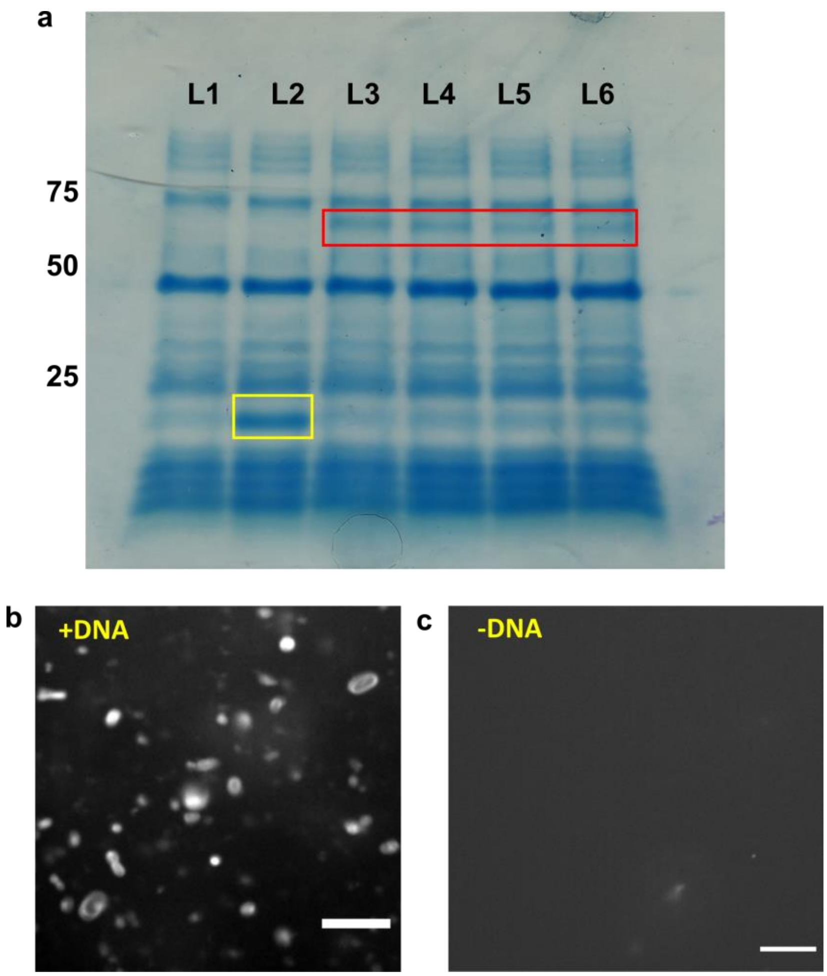

Supplementary Figure 16. Linking gene expression to phospholipid membrane formation. (**a**) SDS-PAGE analysis of FadD10 expression in PURE System. **L1**: No DNA (negative control); **L2**: DHFR DNA (positive control), expressed DHFR band is indicated by yellow box; **L3**: 50 ng FadD10 DNA; **L4**: 100 ng FadD10 DNA; **L5**: 150 ng FadD10 DNA; **L6**: 200 ng FadD10 DNA. All FadD10 bands are indicated in the red box. (**b**) *De novo* vesicle formation in PURExpress® System takes place when the lipid forming precursors (lysolipid **2**, dodecanoic acid, $MgCl_2$ and ATP) are added after expression of FadD10 (+DNA) has taken place at 37 °C. The membranes are stained with 0.1 mol% Texas Red® DHPE. Scale bar represents 10 μm. (**c**) Control experiment showing that when no DNA (encoding FadD10) is added in the beginning, vesicles are not formed (Texas Red® Channel) after the lipid forming precursors are added at 37 °C. Scale bar represents 10 μm.

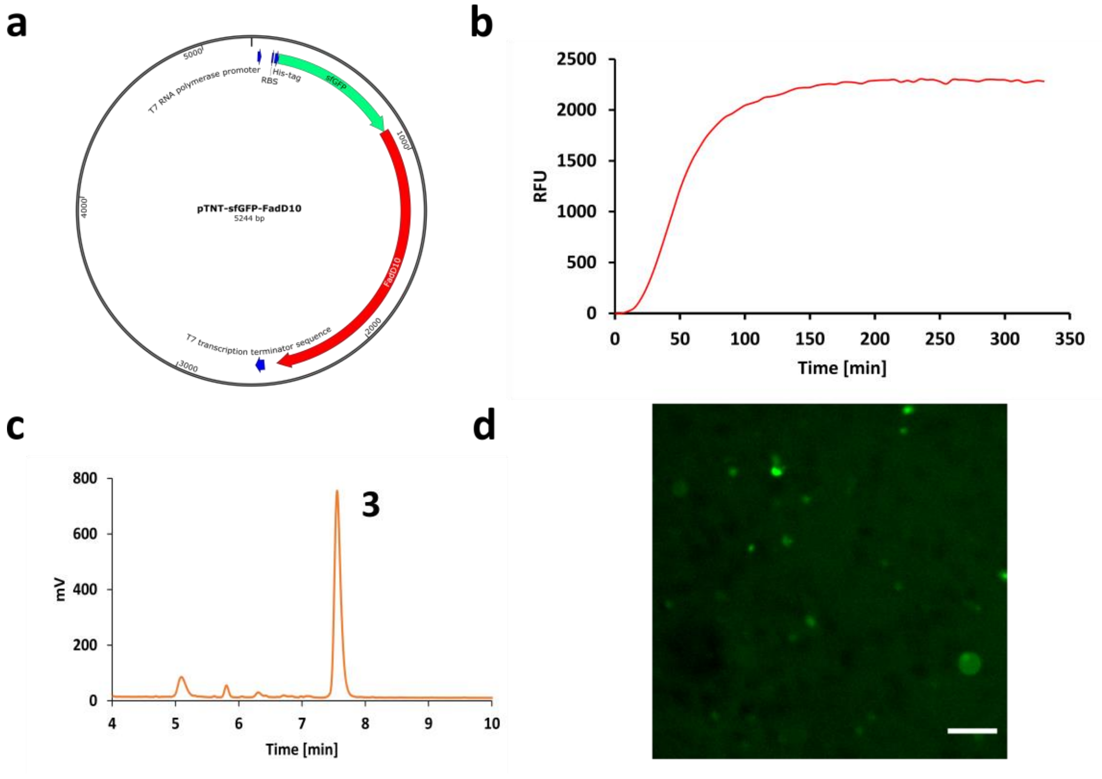

Supplementary Figure 17. *De novo* phospholipid formation mediated by sfGFP-FadD10. (**a**) Map of the plasmid encoding sfGFP-FadD10. **(b**) Monitoring expression of sfGFP-FadD10 in PURExpress® System at 37 ºC by spectrofluorimetry. (**c**) Formation of phospholipid **3** catalyzed by sfGFP-FadD10 in presence of the lipid precursors at 37ºC. (**d**) *De novo* phospholipid membrane formation (from 1 mM lysolipid **2**, 1 mM dodecanoic acid, 3 mM ATP, 9 mM $MgCl_2$) catalyzed by purified sfGFP-FadD10 at pH 8.0. The external proteins were digested by addition of proteinase K. Scale bar represents 5 µm.

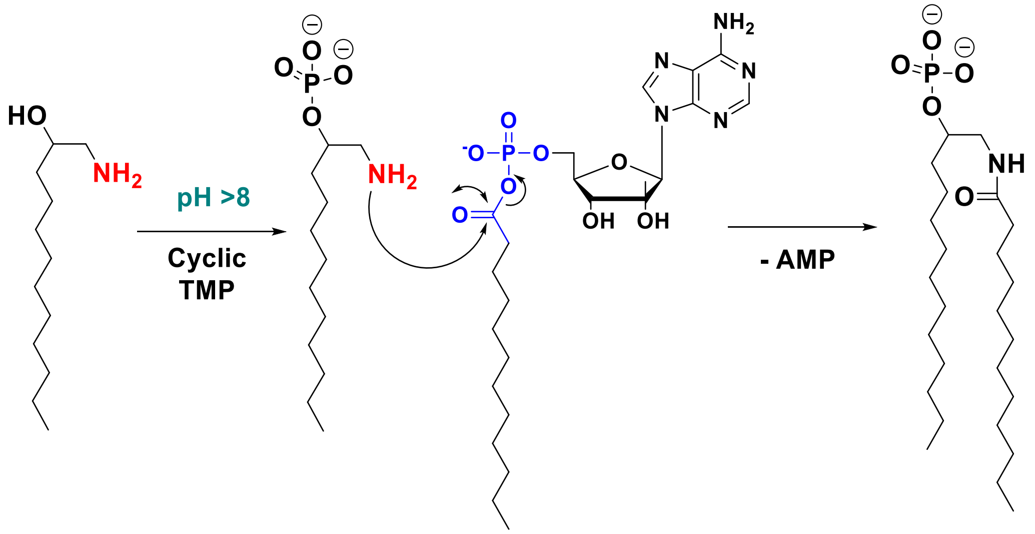

Supplementary Figure 18. Formation of a prebiotically-likely phospholipid analogue. Proposed scheme for reaction between a prebiotically plausible single chain amino-amphiphile[2] and a fatty acyl adenylate leading to the formation of a two-chain phospholipid-like molecule. TMP stands for trimetaphosphate.

a

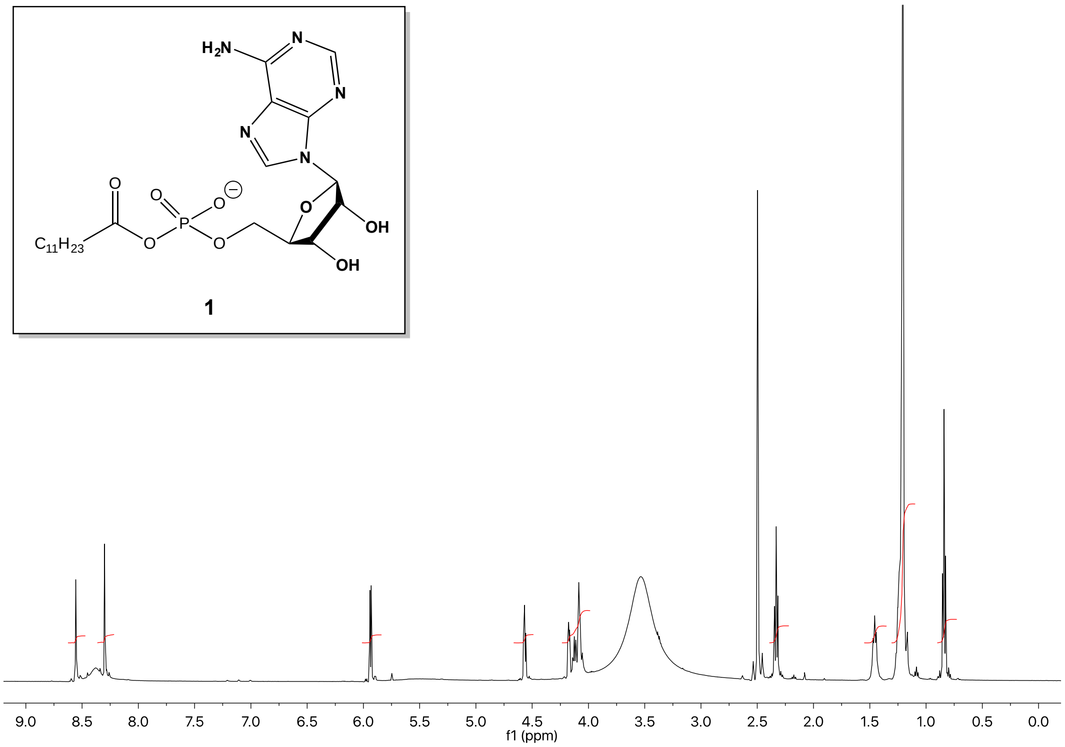

b

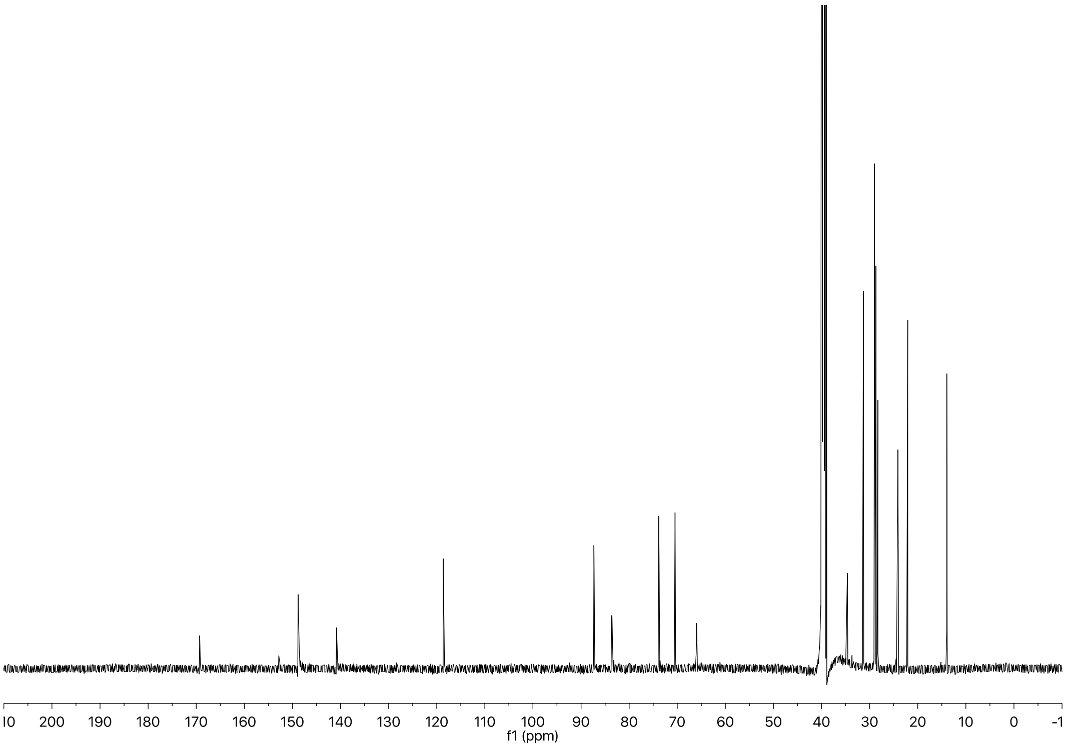

Supplementary Figure 19. NMR spectra of dodecanoyl-AMP (**1**). (**a**) ^{1}H NMR (**b**) ^{13}C NMR in d_6-DMSO.

a

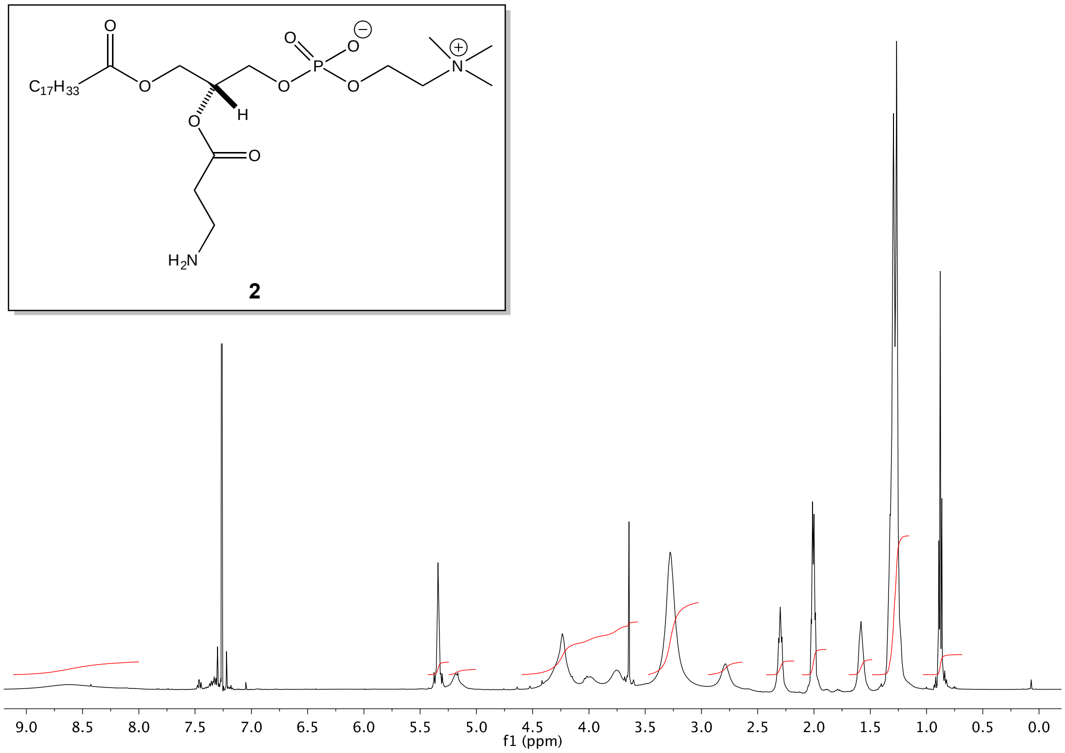

b

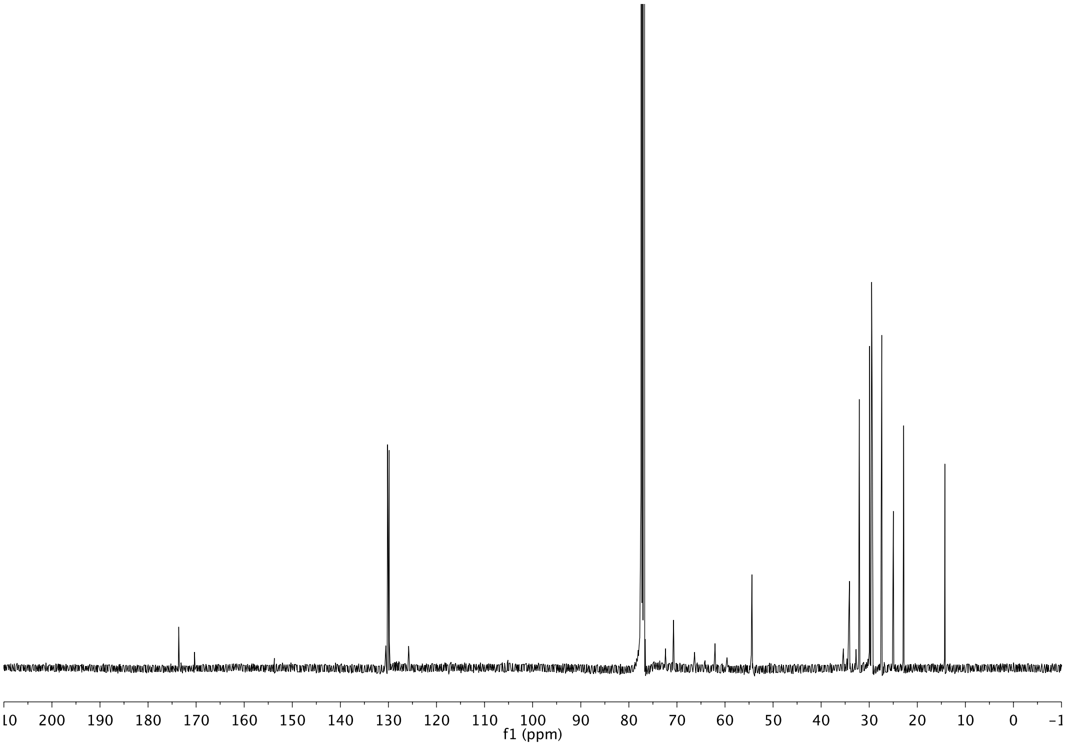

Supplementary Figure 20. NMR spectra of 1-oleoyl-2-(β-Ala)-*sn*-glycero-3-phosphocholine (**2**). (**a**) ^{1}H NMR (**b**) ^{13}C NMR in $CDCl_3$.

a

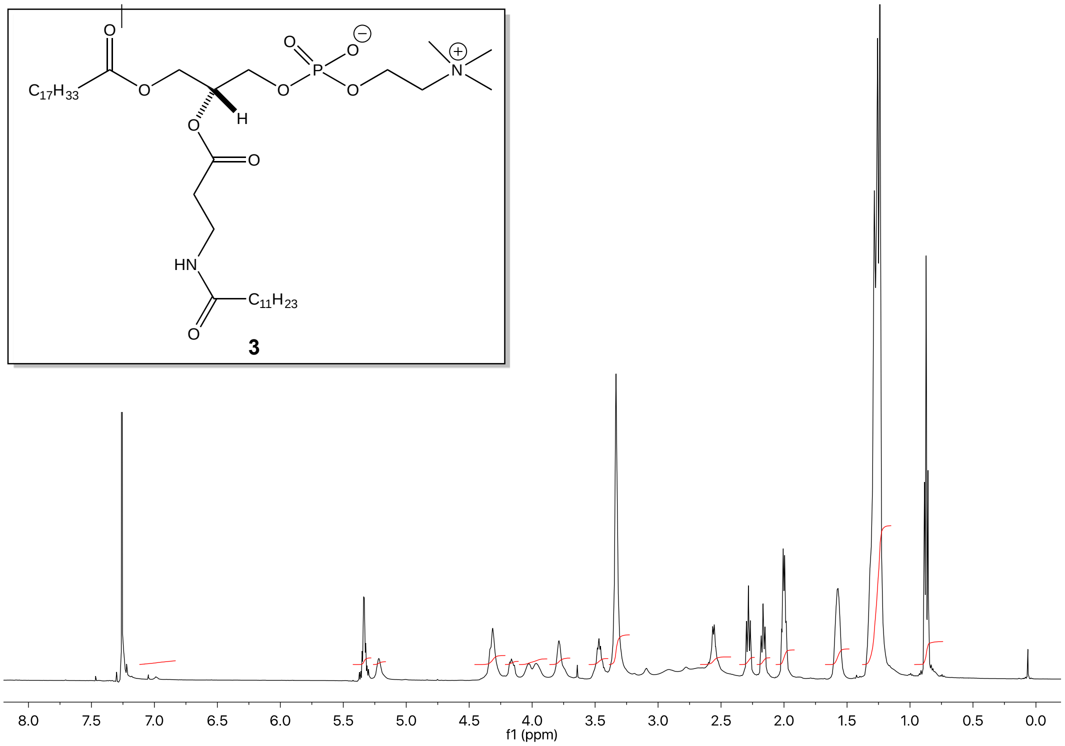

b

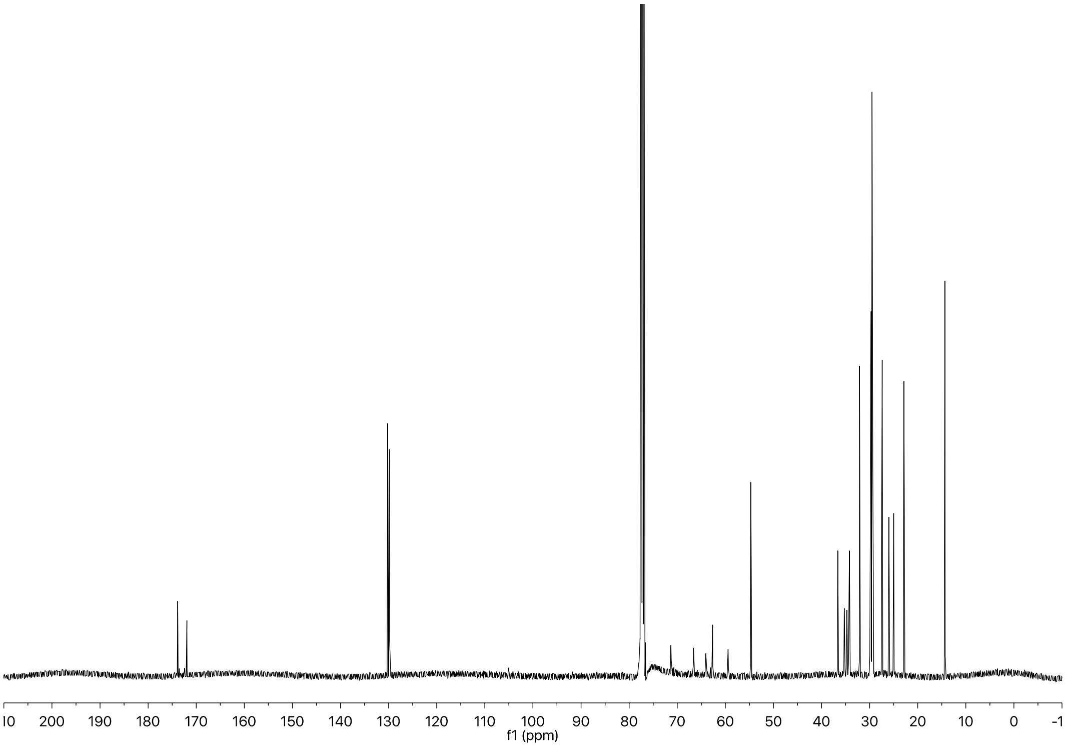

Supplementary Figure 21. NMR spectra of 1-oleoyl-2-[β-Ala-(dodecanoyl)]-*sn*-glycero-3-phosphocholine (**3**). (**a**) ^{1}H NMR (**b**) ^{13}C NMR in $CDCl_3$.

a

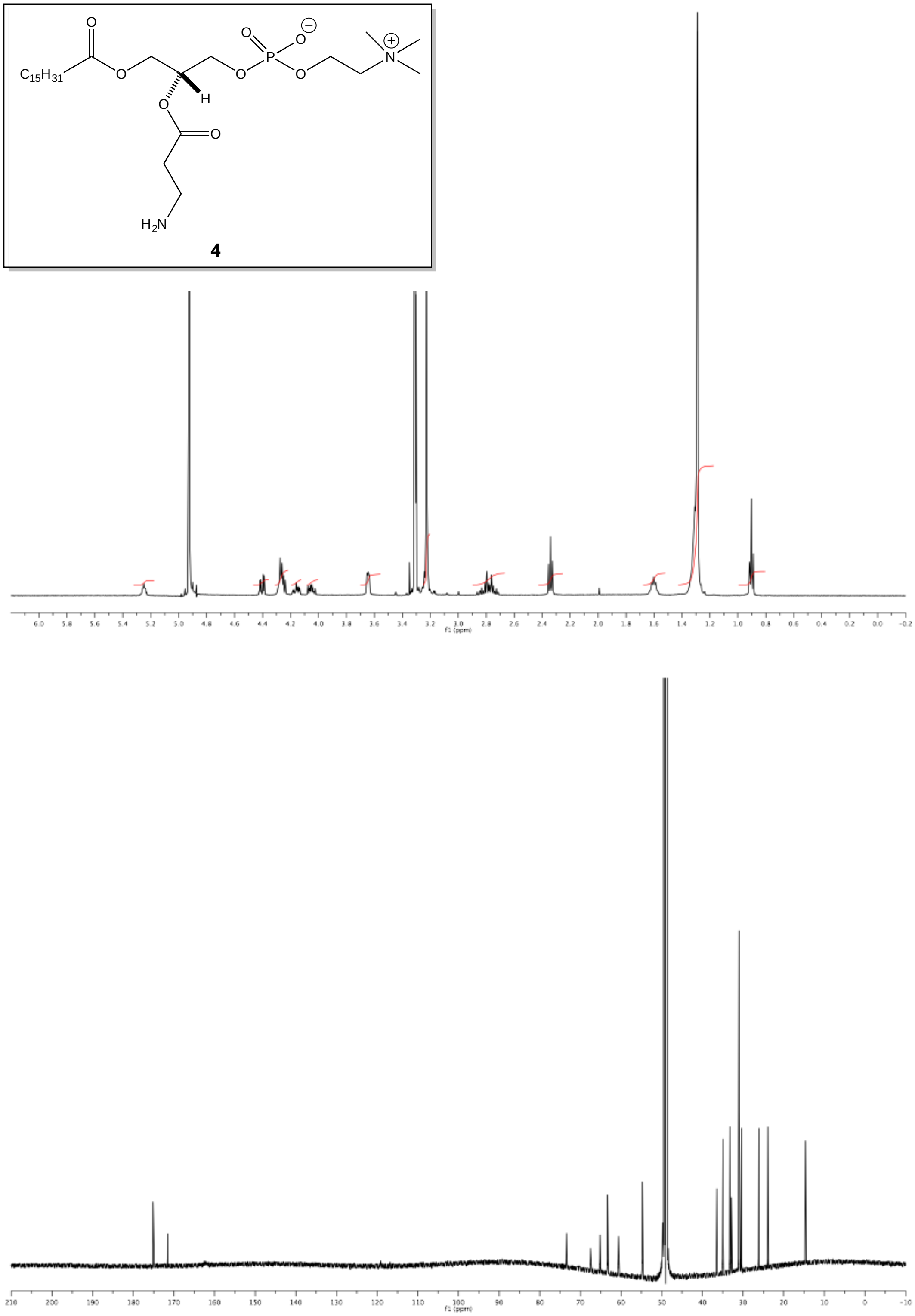

Supplementary Figure 22. NMR spectra of 1-palmitoyl-2-(β-Ala)-*sn*-glycero-3-phosphocholine (**4**). (**a**) ^{1}H NMR (**b**) ^{13}C NMR in CD_3OD.

a

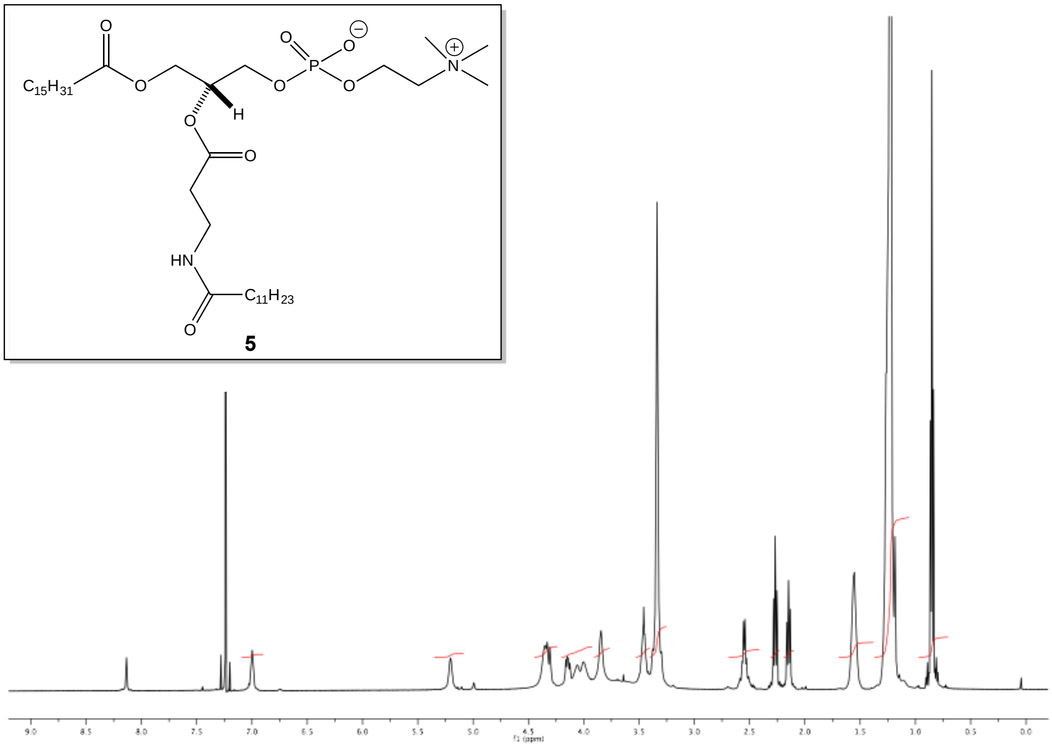

b

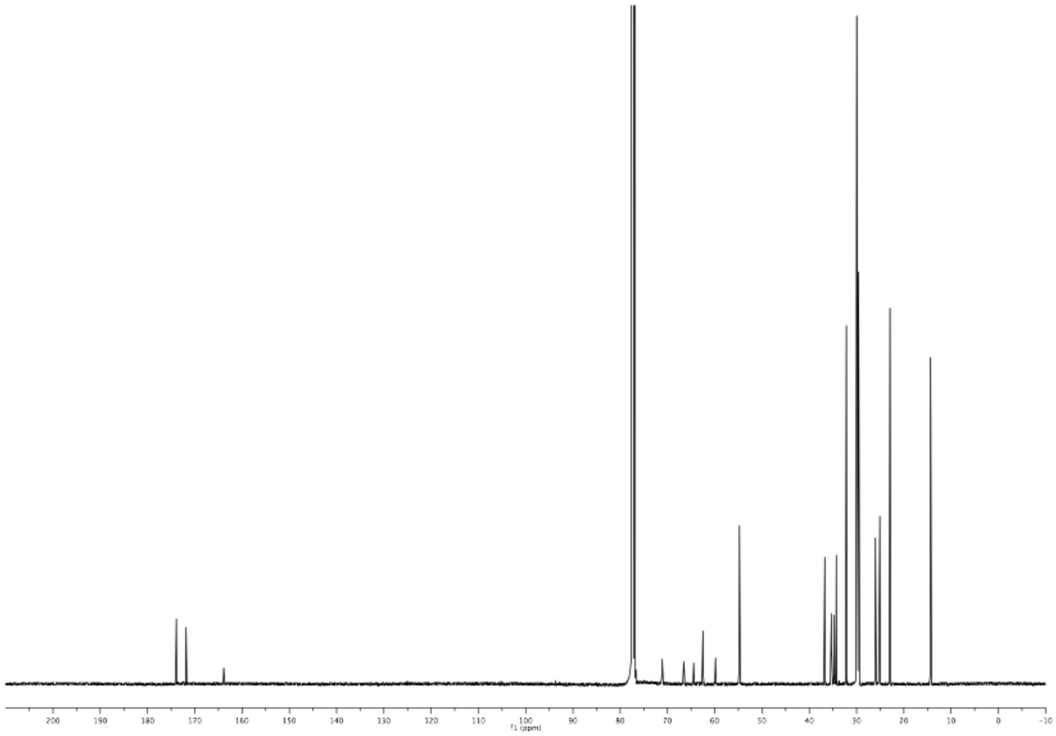

Supplementary Figure 23. NMR spectra of 1-palmitoyl-2-[β-Ala-(dodecanoyl)]-*sn*-glycero-3-phosphocholine (**5**). (**a**) ^{1}H NMR (**b**) ^{13}C NMR in $CDCl_3$.

a

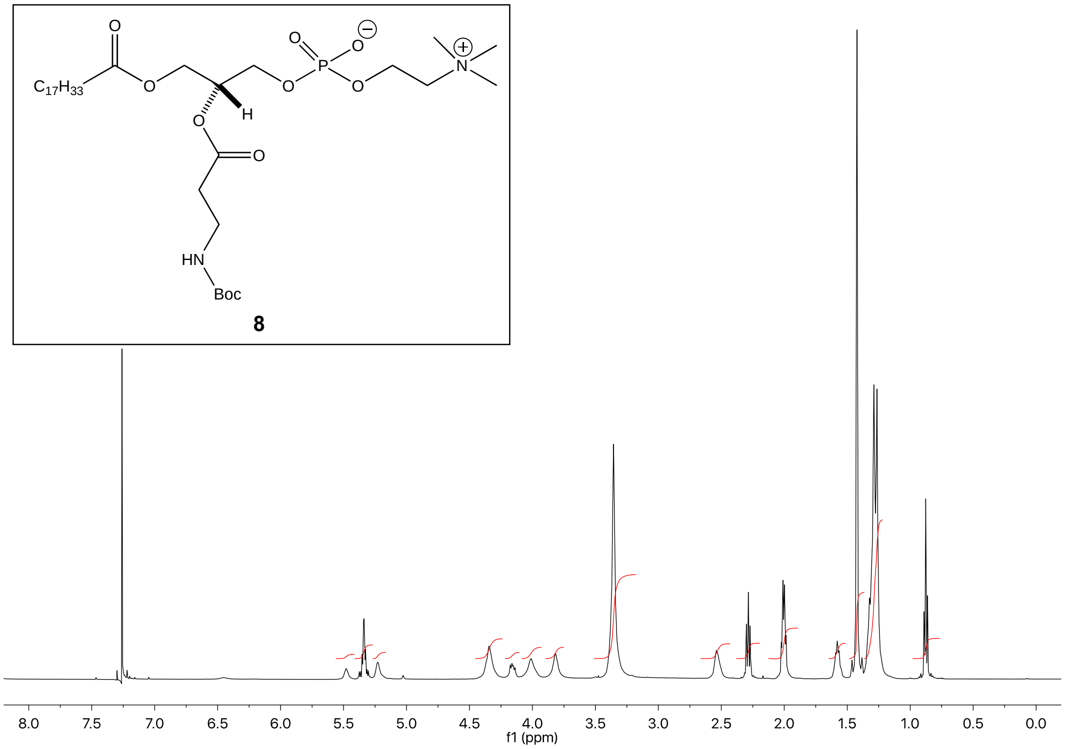

b

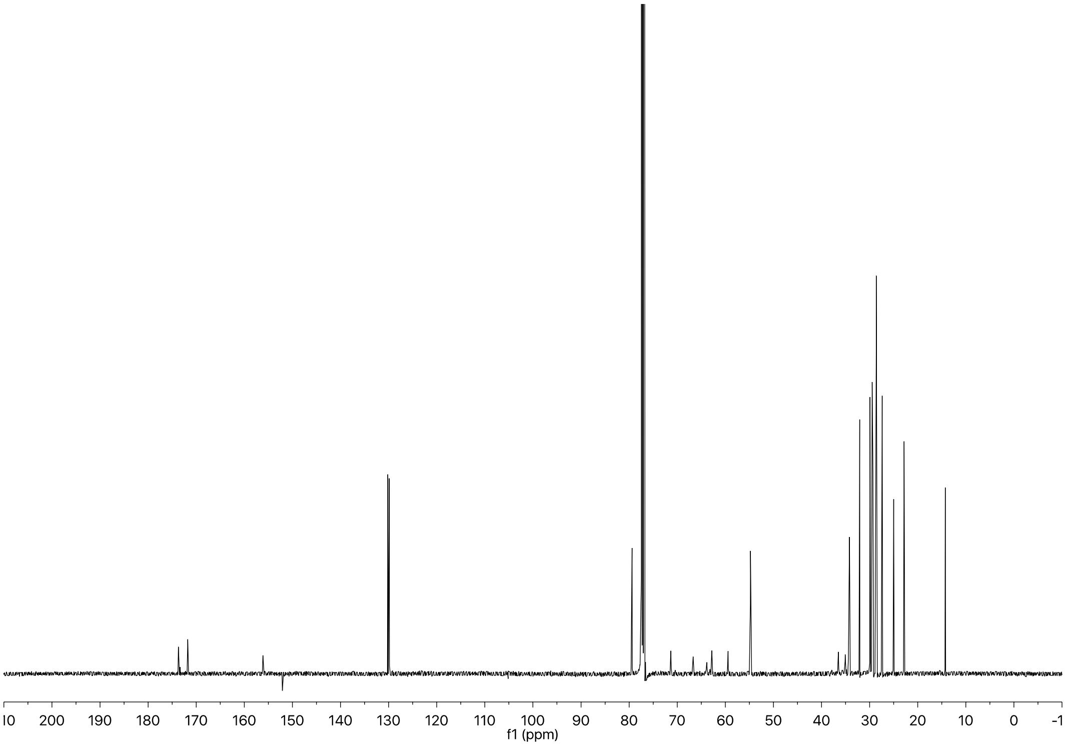

Supplementary Figure 24. NMR spectra of 1-oleoyl-2-(*N*-Boc-β-Ala)-*sn*-glycero-3-phosphocholine (**8**). (**a**) ^{1}H NMR (**b**) ^{13}C NMR in $CDCl_3$.

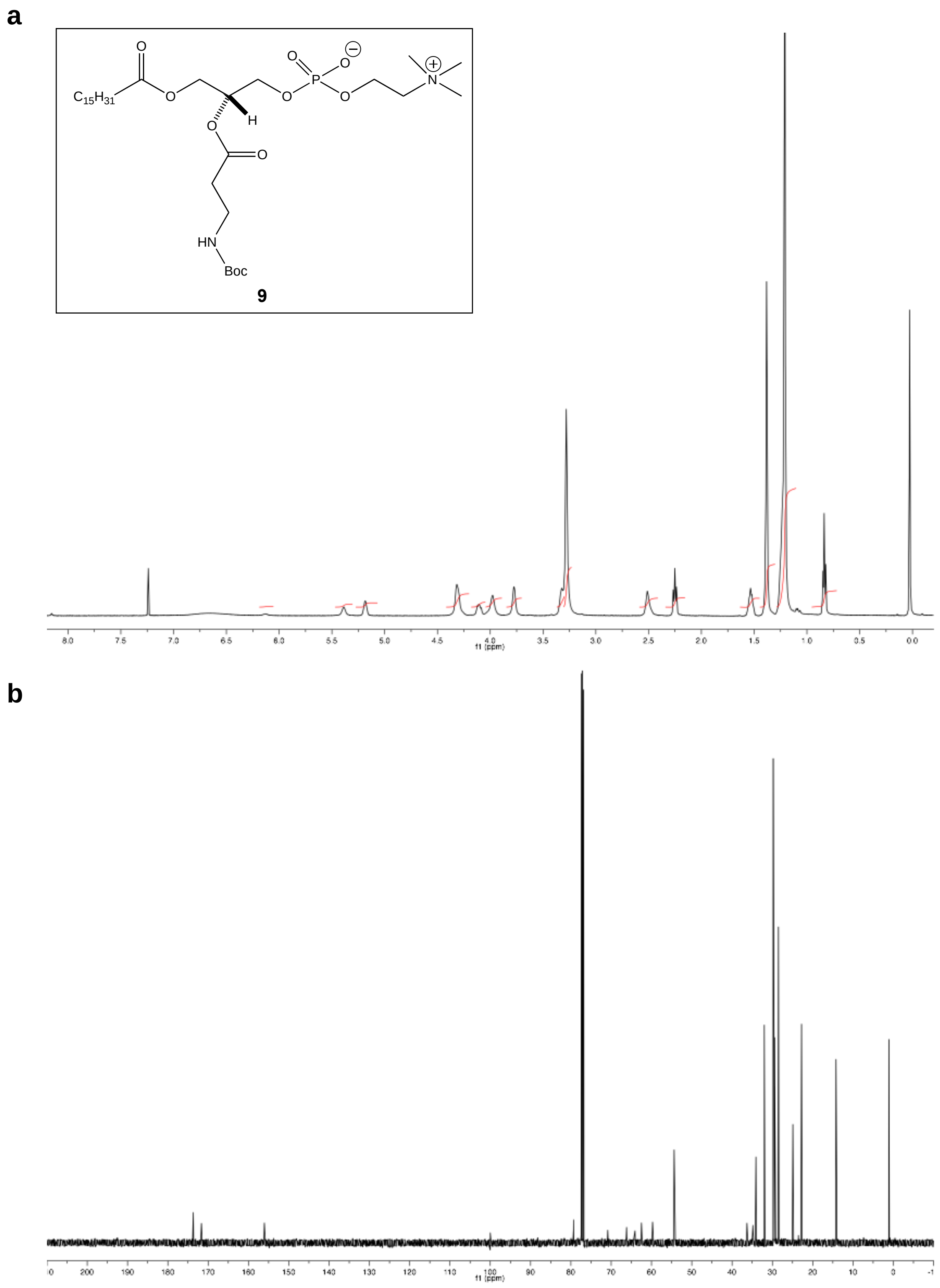

Supplementary Figure 25. NMR spectra of 1-palmitoyl-2-(*N*-Boc-*β*-Ala)-*sn*-glycero-3-phosphocholine (**9**). (**a**) ^{1}H NMR (**b**) ^{13}C NMR in $CDCl_3$.

a

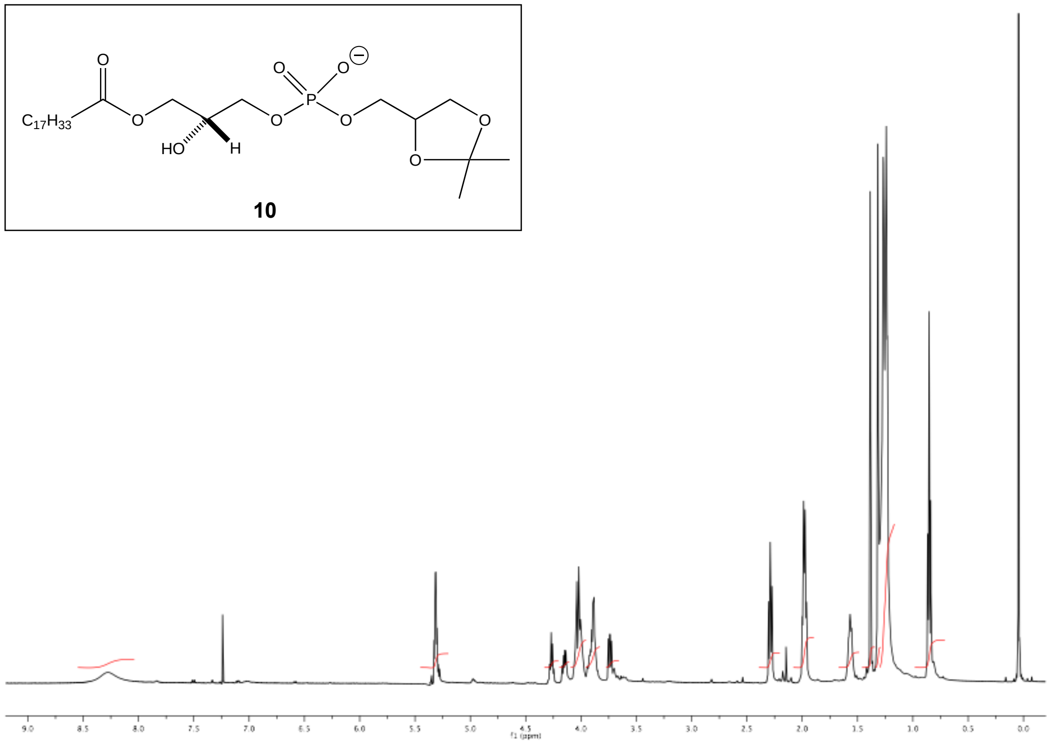

b

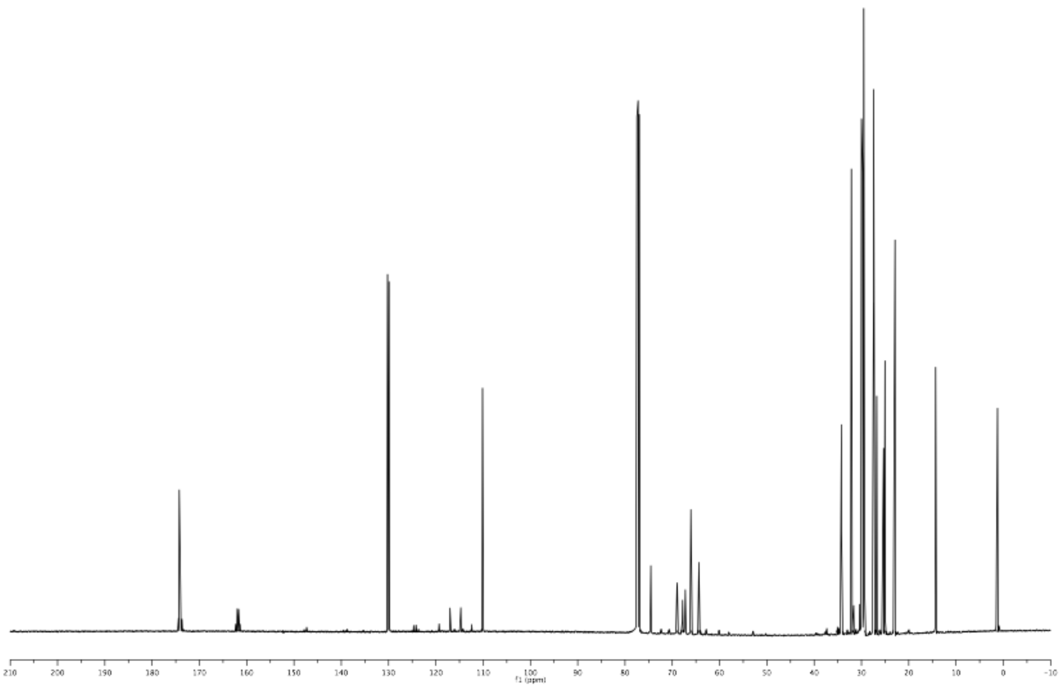

Supplementary Figure 26. NMR spectra of acetonide-protected 1-oleoyl-2-hydroxy-*sn*-glycero-3-phosphoglycerol (**10**). (**a**) ^{1}H NMR (**b**) ^{13}C NMR in $CDCl_3$.

a

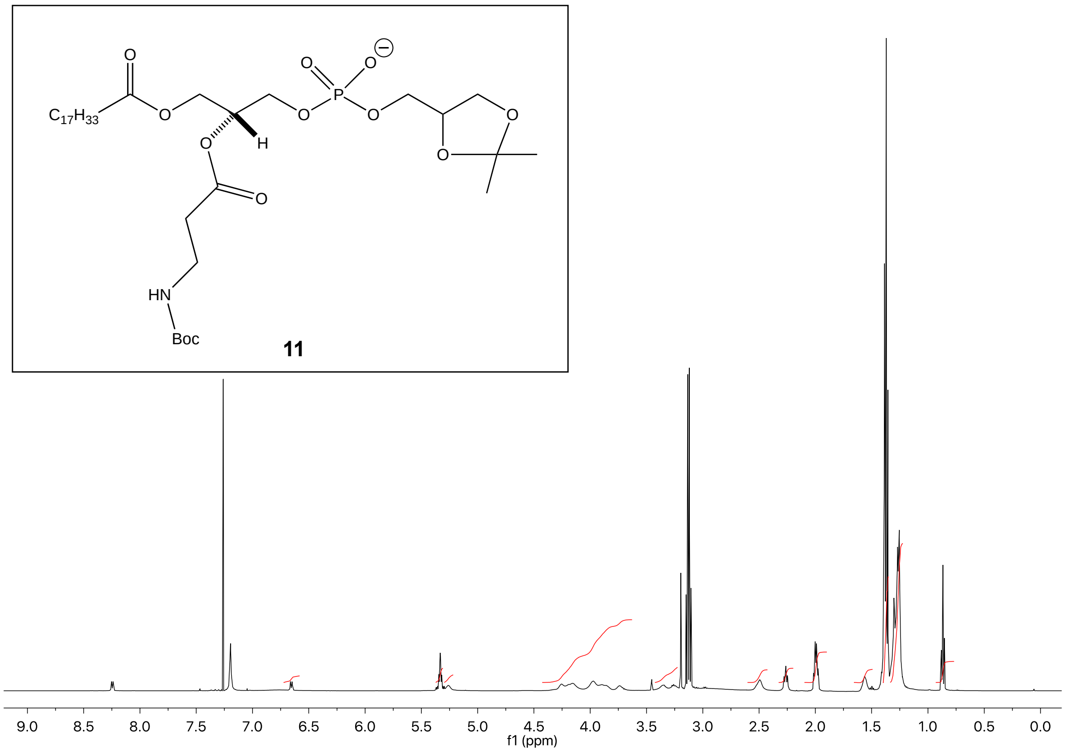

b

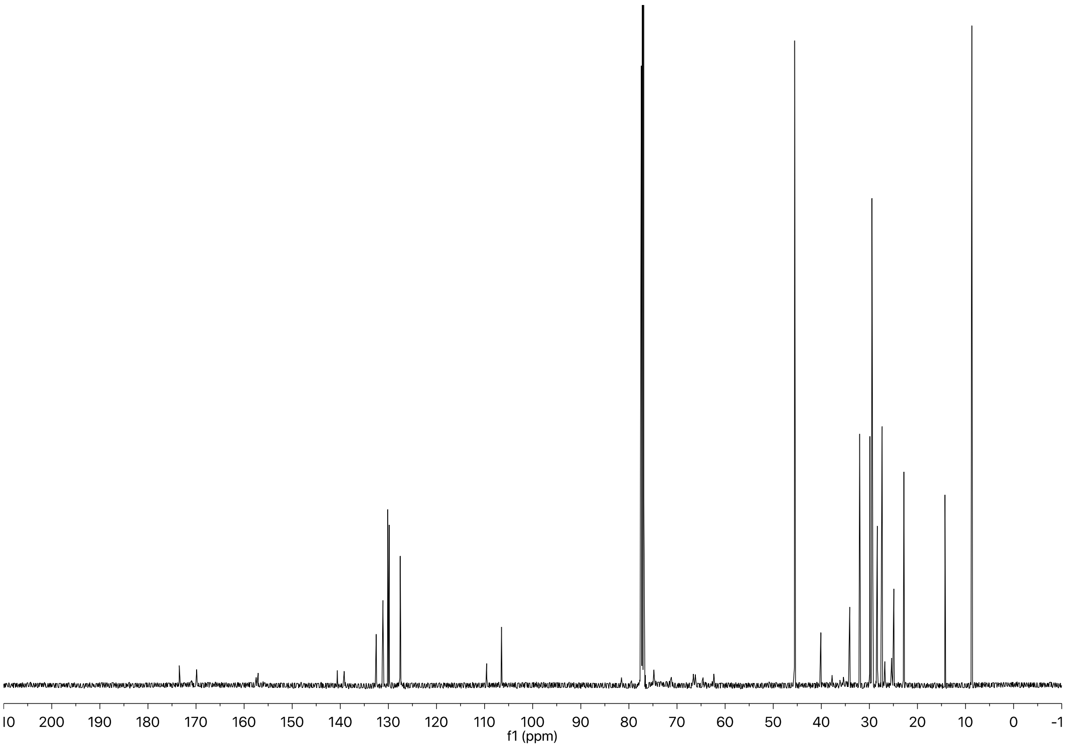

Supplementary Figure 27. NMR spectra of acetonide-protected 1-oleoyl-2-[*N*-Boc-β-Ala]-*sn*-glycero-3-phosphoglycerol (**11**). (**a**) ^{1}H NMR (**b**) ^{13}C NMR in $CDCl_3$.

a

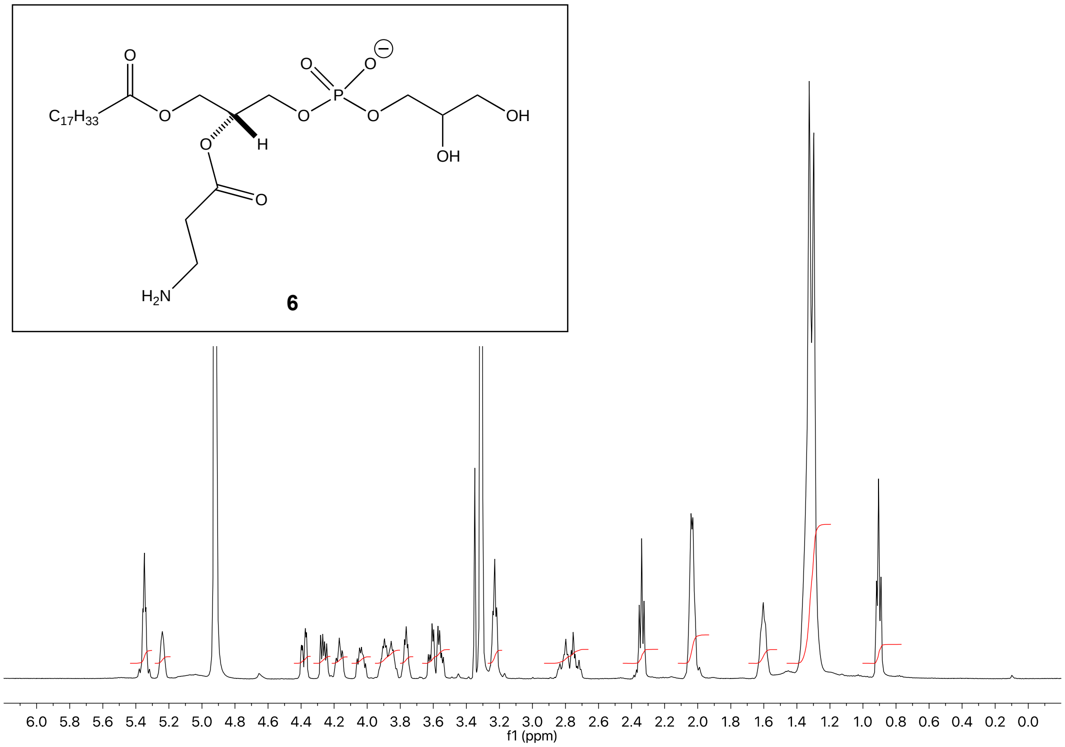

b

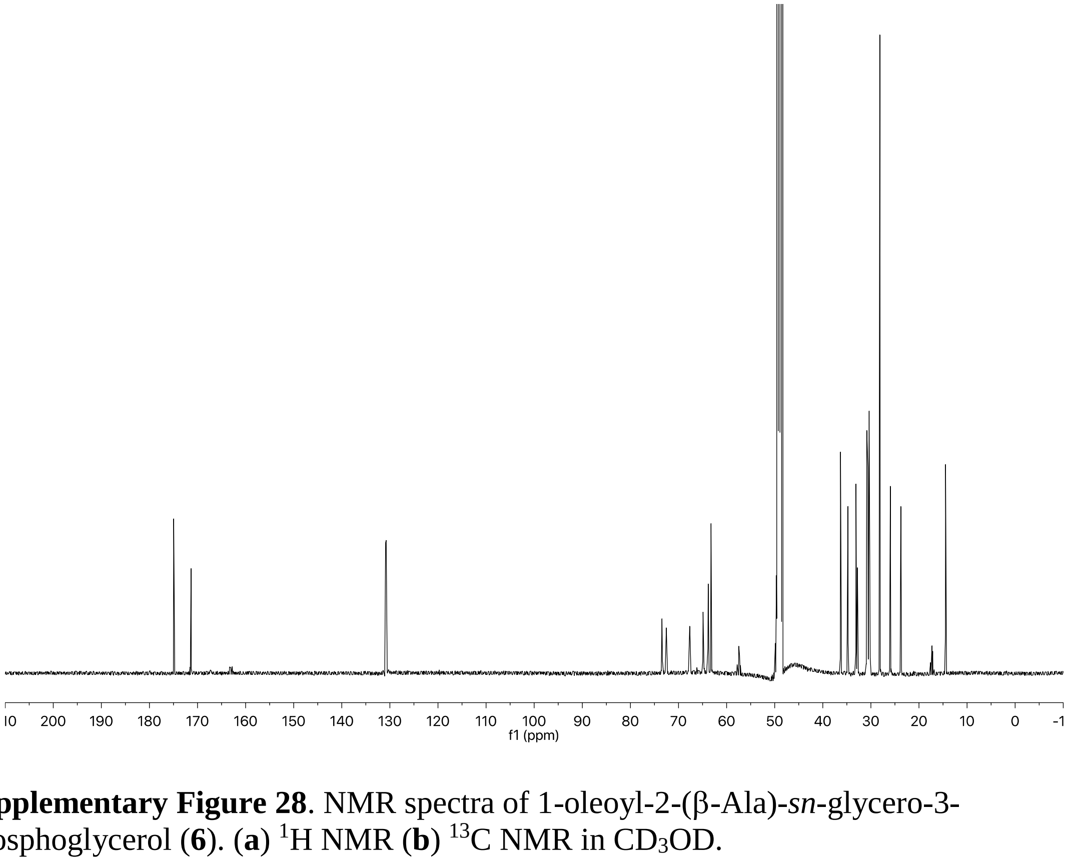

Supplementary Figure 28. NMR spectra of 1-oleoyl-2-(β-Ala)-*sn*-glycero-3-phosphoglycerol (**6**). (**a**) ^{1}H NMR (**b**) ^{13}C NMR in CD_3OD.

Supplementary Tables

Supplementary Table 1. List of primers used in this study

Primer name	Primer sequence
FadD10 Fwd	5´-gatgaactgtacaaaatgggaggaaagaagtttcaagc-3´
FadD10 Rev	5´-cccgggtcgactctagaggtaccattattatcagccacgaacgaccactc-3´
pTNT Rev	5´-ggtacctttctcctctttaatgaattc-3´
pTNT Fwd	5´-taataatggtacctctagagtcgacc-3´
His sfGFP Fwd	5’-accgaattcattaaagaggagaaaggtaccatgcatcaccatcaccatcacatgcgtaaa ggagaagaac-3´
His sfGFP Rev	5´-cttctttcctcccattttgtacagttcatccatacc-3´

Supplementary Methods

UV-Vis and fluorescence measurements

UV-Vis measurements for miscellaneous purposes were recorded on a Thermo Scientific NanoDrop 2000C spectrophotometer. The instrument was blanked with the appropriate solution prior to measurements. Fluorescence measurements were carried out on a Tecan Spark Plate Reader.

Osmometry

Osmolarities were measured using Advanced Micro Osmometer (Advanced Instruments, Model 3300). It is very important that the osmolarities of the vesicle solution and the flow solution are well-matched.

Optical microscopy

Spinning-disk confocal microscopy images were acquired on a Yokagawa spinning disk system (Yokagawa, Japan) built around an Axio Observer Z1 motorized inverted microscope (Carl Zeiss Microscopy GmbH, Germany) with an Evolve 512x512 EMCCD camera (Photometrics, Canada) using ZEN imaging software (Carl Zeiss Microscopy GmbH, Germany). Phase contrast images were taken on an Olympus BX51 microscope.

Cryogenic electron microscopy

Samples were prepared using the commercial environmentally-controlled automated Vitrobot (FEI, Netherlands) at a controlled temperature of 4 ºC and ≈95% humidity[3]. All grid treatments and simple depositions were on the dark/shiny/glossy face of the grid (the side face up during glow discharge). Micrographs were recorded on a helium-cooled FEI Tecnai™ G^2 Polara microscope operating at 300 kV and equipped with a field emission gun (FEG), using an in-house developed cryo-stage that allows for examination of multiple, vitrified samples at either liquid nitrogen or liquid helium temperatures. Images were recorded digitally on a Gatan UltraScan 4000 UHS CCD camera using Digital Micrograph (Gatan, U.K.) in the low-dose imaging mode to minimize beam exposure and electron-beam radiation damage. For image processing, micrographs were digitized in a Zess SCAI scanner with different sampling windows.

Plasmids

The plasmid encoding FadD10 (pDEST17-*Rv0099*) was generously provided by Prof. James Sacchettini (Texas A&M University). Plasmid encoding N-terminal His_6-tagged sfGFP-FadD10 was constructed on a pTNT backbone using standard molecular cloning tools and methods.

Synthesis of 1-oleoyl-2-(*N*-Boc-β-Ala)-*sn*-glycero-3-phosphocholine **(8).** A solution of 1-oleoyl-2-hydroxy-*sn*-glycero-3-phosphocholine (**Lyso $C_{18:1}$ PC-OH**, 14.0 mg, 26.8 μmol), *N*-Boc-β-Ala-OH (12.7 mg, 67.1 μmol), DMAP (15.7 mg, 161.0 μmol) and Et_3N (13.1 μL, 90.9 μmol) in $CDCl_3$ (1 mL) was stirred at rt for 10 min. Then, TCBC

(27.3 μL, 174.4 μmol) was added. After 12 h stirring at rt, H_2O (50 μL) was added to quench the acid chloride, and the solvent was removed under reduced pressure to give a pale yellow solid. The corresponding residue was dissolved in MeOH (500 μL), filtered using a 0.2 μm syringe-driven filter, and the crude solution was purified by HPLC, affording 14.0 mg of **7** as a colorless film [76%, t_R = 8.5 min (Zorbax SB-C18 semi-preparative column, 5% *Phase A* in *Phase B*, 15.5 min)]. ^{1}H NMR ($CDCl_3$, 500.13 MHz, δ): 5.48 (br s, 1H, 1 × NH), 5.41-5.27 (m, 2H, 2 × CH), 5.26-5.16 (m, 1H, 1 × CH), 4.46-4.24 (m, 3H, 1.5× CH_2), 4.22-4.10 (m, 1H, 0.5× CH_2), 4.09-3.93 (m, 2H, 1 × CH_2), 3.90-3.75 (m, 2H, 1 × CH_2), 3.51-3-18 (m, 2H, 1 × CH_2), 3.36 (s, 9H, 3 × CH_3), 2.67-2.43 (m, 2H, 1 × CH_2), 2.29 (t, *J* = 7.6 Hz, 2H, 1 × CH_2), 2.13-1.89 (m, 4H, 1 × CH_2), 1.65-1.51 (m, 2H, 1 × CH_2), 1.42 (s, 9H, 3 × CH_3), 1.37-1.22 (m, 20H, 10 × CH_2), 0.88 (t, *J* = 6.8 Hz, 3H, 1 × CH_3). ^{13}C NMR ($CDCl_3$, 125.77 MHz, δ): 173.7, 171.8, 156.1, 130.2, 129.9, 79.4, 71.3, 66.7, 63.9, 62.8, 59.4, 54.8, 36.5, 35.1, 34.2, 32.1, 29.9, 29.9, 29.7, 29.5, 29.5, 29.4, 29.3, 29.3, 28.6, 27.4, 27.3, 25.0, 22.8, 14.3. MS (ESI-TOF) [m/z (%)]: 693 ($[MH]^+$, 100). HRMS (ESI-TOF) calculated for $C_{34}H_{66}N_2O_{10}P$ ($[MH]^+$) 693.4450, found 693.4446.

Synthesis of 1-oleoyl-2-(β-Ala)-*sn*-glycero-3-phosphocholine **(2)**. A solution of 1-oleoyl-2-(*N*-Boc-β-Ala)-*sn*-glycero-3-phosphocholine (**8**, 10.1 mg, 14.6 μmol) in 0.35 mL of TFA/CH_2Cl_2 (1:1) was stirred at rt for 15 min. After removal of the solvent, the residue was dried under high vacuum for 3 h. Then, the corresponding residue was diluted in MeOH (500 μL), filtered using a 0.2 μm syringe-driven filter, and the crude solution was purified by HPLC, affording 8.4 mg of the lysolipid **2** as a colorless oil [96%, t_R = 4.0 min (Zorbax SB-C18 semi-preparative column, 50% *Phase A* in *Phase B*, 1 min, and then 5% *Phase A* in *Phase B*, 10 min)]. ^{1}H NMR ($CDCl_3$, 500.13 MHz, δ): 8.60 (br s, 2H, 1 × NH_2), 5.40-5.27 (m, 2H, 2 × CH), 5.26-5.06 (m, 1H, 1 × CH), 4.56-3.57 (m, 8H, 4 × CH_2), 3.47-3.02 (m, 2H, 1 × CH_2), 3.27 (s, 9H, 3 × CH_3), 2.94-2.64 (m, 2H, 1 × CH_2), 2.30 (t, *J* = 7.8 Hz, 2H, 1 × CH_2), 2.11-1.89 (m, 4H, 2 × CH_2), 1.69-1.48 (m, 2H, 1 × CH_2), 1.47-1.15 (m, 20H, 10 × CH_2), 0.88 (t, *J* = 6.6 Hz, 3H, 1 × CH_3). ^{13}C NMR ($CDCl_3$, 125.77 MHz, δ): 173.6, 170.3, 130.2, 129.8, 72.4, 70.7, 66.4, 62.1, 59.6, 54.4, 35.4, 34.1, 32.7, 32.1, 29.9, 29.9, 29.7, 29.5, 29.5, 29.4, 29.3, 29.3, 27.4, 27.4, 25.0, 22.8, 14.3. MS (ESI-TOF) [m/z (%)]: 593 ($[MH]^+$, 100). HRMS (ESI-TOF) calculated for $C_{29}H_{58}N_2O_8P$ ($[MH]^+$) 593.3925, found 593.3921.

Synthesis of 1-palmitoyl-2-(*N*-Boc-*β*-Ala)-*sn*-glycero-3-phosphocholine **(9)**. A solution of 1-palmitoyl-2-hydroxy-*sn*-glycero-3-phosphocholine (**Lyso $C_{16:0}$ PC-OH**, 47.5 mg, 95.8 μmol), N-Boc-β-Ala-OH (45.3 mg, 239.5 μmol), DMAP (70.2 mg, 574.8 μmol) and Et_3N (46.7 μL, 335.3 μmol) in $CDCl_3$ (7.5 mL) was stirred at rt for 10 min. Then, TCBC (97.3 μL, 622.7 μmol) was added. After 12 h stirring at rt, H_2O (250 μL) was added to quench the acid chloride, and the solvent was removed under reduced pressure to give a pale yellow solid. The corresponding residue was dissolved in MeOH (1 mL), filtered using a 0.2 μm syringe-driven filter, and the crude solution was purified by HPLC, affording 49.8 mg of **8** as a white foam [78%, t_R = 6.8 min (Zorbax SB-C18 semi-preparative column, 5% *Phase A* in *Phase B*, 15.5 min)]. ^{1}H NMR ($CDCl_3$, 500.13 MHz, δ): 6.15 (br s, 1H, 0.3H, 0.3 × NH), 5.41 (br s, 0.7H, 0.7 × NH), 5.21 (m, 1H, 1 × CH), 4.40-4.26 (m, 3H, 1.5× CH_2), 4.17-4.08 (m, 1H, 0.5× CH_2), 4.07-3.94 (m,

2H, 1 × CH_2), 3.85-3.75 (m, 2H, 1 × CH_2), 3.38-3-31 (m, 2H, 1 × CH_2), 3.30 (s, 9H, 3 × CH_3), 2.59-2.46 (m, 2H, 1 × CH_2), 2.27 (t, J = 7.6 Hz, 2H, 1 × CH_2), 1.61-1.50 (m, 2H, 1 × CH_2), 1.40 (s, 9H, 3 × CH_3), 1.30-1.20 (m, 24H, 12 × CH_2), 0.86 (t, J = 6.8 Hz, 3H, 1 × CH_3). ^{13}C NMR ($CDCl_3$, 125.77 MHz, δ): 173.8, 171.8, 156.1, 79.4, 70.9, 66.3, 64.2, 62.6, 59.8, 54.5, 36.4, 34.9, 34.2, 32.1, 29.9, 29.9, 29.8, 29.7, 29.5, 29.5, 29.3, 28.6, 25.0, 22.9, 14.3. MS (ESI-TOF) [m/z (%)]: 689 ($[M+Na]^+$, 100). HRMS (ESI-TOF) calculated for $C_{32}H_{63}N_2O_{10}PNa$ ($[M+Na]^+$) 689.4113, found 689.4114.

Synthesis of 1-palmitoyl-2-(β-Ala)-*sn*-glycero-3-phosphocholine **(4)**. A solution of 1-palmitoyl-2-(*N*-Boc-β-Ala)-*sn*-glycero-3-phosphocholine (**9**, 9.0 mg, 13.5 μmol) in 1 mL of TFA/CH_2Cl_2 (1:1) was stirred at rt for 15 min. After removal of the solvent, the residue was dried under high vacuum for 3 h. Then, the corresponding residue was diluted in MeOH (500 μL), filtered using a 0.2 μm syringe-driven filter, and the crude solution was purified by HPLC, affording 7.2 mg of the lysolipid **4** as a colorless oil [80%, t_R = 8.6 min (Zorbax SB-C18 semi-preparative column, 50% *Phase A* in *Phase B*, 5 min, and then 5% Phase A in Phase B, 10 min)]. ^{1}H NMR (CD_3OD, 500.13 MHz, δ): 5.19 (m, 1H, 1 × CH), 4.37-4.32 (dd, 1H, J_1 = 3.9 Hz, J_2 = 12.1 Hz, 0.5 × CH_2), 4.26-4.16 (m, 3H, 1.5× CH_2), 4.14-4.06 (m, 1H, 0.5 × CH_2), 4.03-3.95 (m, 1H, 0.5 × CH_2), 3.61-3.56 (m, 2H, 1 × CH_2), 3.22-3.13 (m, 2H, 1 × CH_2), 3.17 (s, 9H, 3 × CH_3), 2.82-2.65 (m, 2H, 1 × CH_2), 2.28 (t, J = 7.4 Hz, 2H, 1 × CH_2), 1.59-1.50 (m, 2H, 1 × CH_2), 1.30-1.20 (m, 24H, 12 × CH_2), 0.85 (t, J = 6.8 Hz, 3H, 1 × CH_3). ^{13}C NMR (CD_3OD, 125.77 MHz, δ): 175.1, 171.5, 73.4, 67.5, 65.2, 63.3, 60.6, 54.8, 36.4, 34.9, 33.2, 32.8, 31.0, 31.0, 31.0, 31.0, 30.9, 30.9, 30.8, 30.7, 30.6, 30.4, 26.1, 23.9, 14.6. MS (ESI-TOF) [m/z (%)]: 567 ($[MH]^+$, 100). HRMS (ESI-TOF) calculated for $C_{27}H_{56}N_2O_8P$ ($[MH]^+$) 567.3769, found 567.3770.

Synthesis of acetonide-protected 1-oleoyl-2-hydroxy-*sn*-glycero-3phosphoglycerol (10)[4]. A suspension of 1-oleoyl-2-hydroxy-*sn*-glycero-3-phospho-(1´-rac-glycerol) (sodium salt) (**Lyso $C_{18:1}$ PG-OH**, 50.0 mg, 93.9 μmol) in acetone (650 µL) was subsequently treated with 2,2-dimethoxypropane (DMP, 40 mL) and a catalytic amount of TFA (25 µL). After 5 min stirring at rt, $CHCl_3$ (40 mL) was added until the lysolipid was fully dissolved. The reaction mixture was stirred for 60 h at rt. After evaporation of solvent under reduced pressure, the residue was dried *in vacuo* for 30 min to remove the TFA. The product was then re-dissolved in dry $CHCl_3$ (10 mL) (do not use wet $CHCl_3$ as this will react with the acetonide moiety), and the solvent was removed by rotary evaporation. This was repeated a total of three times to remove trace TFA in the oily residue. The corresponding residue was dried *in vacuo* for 5 h to give a pale yellow oil, which was used without further purification. ^{1}H NMR ($CDCl_3$, 500.13 MHz, δ): 8.28 (br s, 1H, 1 × OH), 5.45-5.20 (m, 2H, 2 × CH), 4.33-4.21 (m, 1H, 1 × CH), 4.19-4.10 (m, 1H, 0.5 × CH_2), 4.09-3.96 (m, 4H, 2 × CH_2), 3.95-3.83 (m, 3H, 1.5 × CH_2), 3.77-3.66 (m, 1H, 1 × CH), 2.29 (t, J = 7.5 Hz, 2H, 1 × CH_2), 2.08-1.90 (m, 4H, 2 × CH_2), 1.67-1.49 (m, 2H, 1 × CH_2), 1.38 (s, 3H, 1 × CH_3), 1.32 (s, 3H, 1 × CH_3), 1.30-1.17 (m, 20H, 10 × CH_2), 0.85 (t, J = 6.9 Hz, 3H, 1 × CH_3). ^{13}C NMR ($CDCl_3$, 125.77 MHz, δ): 174.3, 130.2, 129.9, 110.1, 74.5, 69.0, 67.8, 67.2, 66.1, 64.4, 34.2, 32.1, 30.0, 29.9, 29.7, 29.5, 29.5, 29.5, 29.4, 29.4, 27.4, 27.4, 26.8, 25.3, 25.0, 22.9, 14.3. MS (ESI-TOF) [m/z (%)]:

549 ([M]$^-$, 100). HRMS (ESI-TOF) calculated for $C_{27}H_{50}O_9P$ ([M]$^-$) 549.3198, found 549.3193.

Synthesis of acetonide-protected 1-oleoyl-2-[*N*-Boc-β-Ala]-*sn*-glycero-3 phospho-glycerol **(11)**. A solution of acetonide-protected 1-oleoyl-2-hydroxy-*sn*-glycero-3-phosphoglycerol (**10**, 40.0 mg, 69.9 µmol), *N*-Boc-β-Ala-OH (33.1 mg, 174.7 µmol), DMAP (51.2 mg, 419.4 µmol) and Et_3N (34 µL) in $CDCl_3$ (2.5 mL) was stirred for 10 min at rt. Then, 2,4,6-trichlorobenzoyl chloride (TCBC, 71 µL) was added and the reaction was stirred for 12 h at rt. Afterwards, H_2O (75 µL) was added to the reaction mixture to quench the acid chloride, and the solvent was removed by rotary evaporation to give a yellow oil. The crude was dissolved in 500 µL of $CHCl_3$ and purified by flash column chromatography (0-10% MeOH in $CHCl_3$), affording 43.1 mg of **11** as a pale yellow oil [83%, R_f = 0.17 (10% MeOH in $CHCl_3$)]. ^{1}H NMR ($CDCl_3$, 500.13 MHz, δ): 6.66 (d, *J* = 7.3 Hz, 1H, 1 × NH), 5.37-5.31 (m, 2H, 2 × CH), 5.30-5.21 (m, 1H, 1 × CH), 4.43-3.62 (m, 9H, 4 × CH_2 + 1 × CH), 3.43-3.20 (m, 2H, 1 × CH_2), 2.60-2.43 (m, 2H, 1 × CH_2), 2.26 (t, *J* = 7.6 Hz, 2H, 1 × CH_2), 2.00 (q, 4H, *J* = 6.8 Hz, 2 × CH_2), 1.66-1.49 (m, 2H, 1 × CH_2), 1.40-1.35 (m, 15H, 5 × CH_3), 1.33-1.22 (m, 20H, 10 × CH_2), 0.87 (t, *J* = 7.0 Hz, 3H, 1 × CH_3). ^{13}C NMR ($CDCl_3$, 125.77 MHz, δ): 173.5, 169.9, 157.1, 130.1, 129.8, 106.7, 81.6, 74.8, 71.2, 66.6, 66.2, 64.6, 62.3, 40.1, 34.1, 32.0, 29.9, 29.9, 29.7, 29.5, 29.4, 29.3, 29.2, 28.5, 28.4, 27.4, 27.3, 26.8, 25.4, 24.9, 22.8, 14.3. MS (ESI-TOF) [m/z (%)]: 744 ([M+Na]$^+$, 100), 722 ([MH]$^+$, 46). HRMS (ESI-TOF) calculated for $C_{35}H_{64}NO_{12}PNa$ ([M +Na]$^+$) 744.4058, found 744.4056.

Synthesis of 1-oleoyl-2-(β-Ala)-*sn*-glycero-3-phosphoglycerol **(6)**. A solution of acetonide-protected 1-oleoyl-2-[*N*-Boc-β-Ala]-*sn*-glycero-3-phosphoglycerol (**11**, 10.0 mg, 13.45 µmol) in 1 mL of TFA/CH_2Cl_2/TES (0.45:0.45:0.1) was stirred at rt for 30 min. After removal of the solvent, the residue was dried under high vacuum for 3 h. Then, the corresponding residue was diluted in MeOH (500 µL), filtered using a 0.2 µm syringe-driven filter, and the crude solution was purified by HPLC, affording 5.2 mg of the lysolipid **6** as a colorless oil [67%, t_R = 11.2 min (Zorbax SB-C18 semipreparative column, 50% *Phase A* in *Phase B*, 5 min, and then 5% *Phase A* in *Phase B*, 10 min)]. ^{1}H NMR (MeOD, 500.13 MHz, δ): 5.44-5.30 (m, 2H, 2 × CH), 5.29-5.19 (m, 1H, 1 × CH), 4.44-4.34 (m, 1H, 0.5 × CH_2), 4.33-4.22 (m, 1H, 0.5 × CH_2), 4.21-4.12 (m, 1H, 0.5 × CH_2), 4.09-3.98 (m, 1H, 0.5 × CH_2), 3.95-3.81 (m, 2H, 1 × CH_2), 3.80-3.72 (m, 1H, 1 × CH), 3.67-3.50 (m, 2H, 1 × CH_2), 3.23 (t, *J* = 6.2 Hz, 2H, 1 × CH_2), 2.93-2.66 (m, 2H, 1 × CH_2), 2.34 (t, *J* = 7.5 Hz, 2H, 1 × CH_2), 2.12-1.93 (m, 4H, 2 × CH_2), 1.69-1.52 (m, 2H, 1 × CH_2), 1.46-1.19 (m, 20H, 10 × CH_2), 0.90 (t, *J* = 6.8 Hz, 3H, 1 × CH_3). ^{13}C NMR (MeOD, 125.77 MHz, δ): 175.0, 171.3, 130.9, 130.8, 73.4, 72.5, 67.7, 64.9, 63.8, 63.2, 36.3, 34.8, 33.1, 32.8, 30.9, 30.8, 30.6, 30.5, 30.4, 30.3, 30.2, 30.2, 28.1, 26.0, 23.8, 14.5. MS (ESI-TOF) [m/z (%)]: 582 ([MH]$^+$, 100). HRMS (ESI-TOF) calculated for $C_{27}H_{53}NO_{10}P$ ([MH]$^+$) 582.3402, found 582.3403.

Synthesis of 1-oleoyl-2-[β-Ala-(dodecanoyl)]-*sn*-glycero-3-phosphocholine **(3)**. 1-oleoyl-2-(β-Ala)-*sn*-glycero-3-phosphocholine (**2**, 5.00 mg, 8.44 µmol) was treated with 844 µL of a 10 mM solution of dodecanoyl-AMP (**1**, 4.47 mg, 8.44 µmol) in 100 mM HEPES pH 7.5 buffer, and stirred at rt. After 2 h, the corresponding mixture was concentrated and

dried *in vacuo*. The resulting white residue was dissolved in MeOH (800 µL) and filtered using a 0.2 µm syringe-driven filter, and the crude solution was purified by HPLC, affording 5.2 mg of the amidophospholipid **3** as a colorless film [80%, t_R = 8.5 min (Zorbax SB-C18 semi-preparative column, *Phase B*, 15.5 min)]. ^{1}H NMR ($CDCl_3$, 500.13 MHz, δ): 6.98 (br s, 1H, 1 × NH), 5.43-5.27 (m, 2H, 1 × CH), 5.26-5.16 (m, 1H, 1 × CH), 4.46-4.21 (m, 3H, 1.5 × CH_2), 4.21-4.16 (m, 1H, 0.5 × CH_2), 4.10-3.88 (m, 2H, 1 × CH_2), 3.86-3.70 (m, 2H, 1 × CH_2), 3.55-3.40 (m, 2H, 1 × CH_2), 3.33 (s, 9H, 3 × CH_3), 2.67-2.42 (m, 2H, 1 × CH_2), 2.28 (t, *J* = 7.7 Hz, 2H, 1 × CH_2), 2.17 (t, *J* = 7.3 Hz, 2H, 1 × CH_2), 2.07-1.92 (m, 4H, 2 × CH_2), 1.67-1.48 (m, 4H, 2 × CH_2), 1.38-1.15 (m, 36H, 18 × CH_2), 0.87 (t, *J* = 6.8 Hz, 6H, 2 × CH_3). ^{13}C NMR ($CDCl_3$, 125.77 MHz, δ): 174.0, 173.9, 171.9, 130.2, 129.8, 71.3, 66.6, 64.1, 62.6, 59.4, 54.7, 36.6, 35.3, 34.7, 34.2, 32.1, 32.1, 29.9, 29.9, 29.9, 29.8, 29.8, 29.7, 29.7, 29.6, 29.6, 29.5, 29.4, 29.4, 29.3, 27.4, 27.4, 26.0, 25.0, 22.9, 22.9, 14.3. MS (ESI-TOF) [m/z (%)]: 775 ([MH]$^+$, 100). HRMS (ESI-TOF) calculated for $C_{41}H_{79}N_2O_9P$ ([MH]$^+$) 775.5596, found 775.5594.

<u>Synthesis of 1-palmitoyl-2-[β-Ala-(dodecanoyl)]-*sn*-glycero-3-phosphocholine (**5**)</u>. 1-palmitoyl-2-(β-Ala)-*sn*-glycero-3-phosphocholine (**4**, 4.25 mg, 7.50 µmol) was treated with 750 µL of a 10 mM solution of dodecanoyl-AMP (**1**, 3.97 mg, 7.50 µmol) in 100 mM HEPES pH 7.5 buffer, and stirred at rt. After 2 h, the corresponding mixture was concentrated and dried *in vacuo*. The resulting white residue was dissolved in MeOH (750 µL) and filtered using a 0.2 µm syringe-driven filter, and the crude solution was purified by HPLC, affording 5.3 mg of the amidophospholipid **5** as a colorless film [94%, t_R = 8.3 min (Zorbax SB-C18 semi-preparative column, *Phase B*, 15.5 min)]. ^{1}H NMR ($CDCl_3$, 500.13 MHz, δ): 7.00 (br s, 1H, 1 × NH), 5.35-5.09 (m, 1H, 1 × CH), 4.45-4.24 (m, 3H, 1.5 × CH_2), 4.20-3.93 (m, 3H, 1.5 × CH_2), 3.91-3.77 (m, 2H, 1 × CH_2), 3.53-3.41 (m, 2H, 1 × CH_2), 3.34 (s, 9H, 3 × CH_3), 2.69-2.42 (m, 2H, 1 × CH_2), 2.27 (t, *J* = 7.7 Hz, 2H, 1 × CH_2), 2.15 (t, *J* = 7.5 Hz, 2H, 1 × CH_2), 1.69-1.38 (m, 4H, 2 × CH_2), 1.37-1.06 (m, 40H, 20 × CH_2), 0.85 (t, *J* = 6.7 Hz, 6H, 2 × CH_3). ^{13}C NMR ($CDCl_3$, 125.77 MHz, δ): 174.0, 173.9, 171.9, 71.2, 66.5, 64.4, 62.5, 59.8, 54.8, 36.7, 35.3, 34.7, 34.3, 32.1, 29.9, 29.9, 29.9, 29.9, 29.8, 29.8, 29.7, 29.6, 29.6, 29.5, 29.4, 26.0, 25.1, 22.9, 14.4. MS (ESI-TOF) [m/z (%)]:749 ([M+H]$^+$, 100). HRMS (ESI-TOF) calculated for $C_{39}H_{78}N_2O_9P$ ([M+H]$^+$) 749.5439, found 749.5437.

<u>Stability study of dodecanoyl-AMP (**1**)</u>

Two 1 mM solutions of the adenylate **1** in 50 mM HEPES pH 7.5 buffer, one containing no Mg^{2+} and the other one containing 10 mM Mg^{2+} (as $MgCl_2$), were kept in a 37 ºC incubator. Then, 10 µL aliquots were taken out from each solution and injected into HPLC at various times (0, 3, 6 and 9 h). The integrated HPLC peak areas (260 nm) for **1** were measured. The experiments were repeated in triplicate and bar graphs were plotted.

<u>*De novo* phospholipid synthesis: *In situ* vesicle formation</u>

In a typical *in situ* vesicle formation experiment, phospholipid **3** was synthesized from 1 mM solutions of each of adenylate **1** and lysolipid **2** in 50 mM HEPES pH 7.5 or 8.0 at 37 ºC. Turbidity appeared after about 15-20 min and a small aliquot (~2 µL) was placed on a glass slide and covered with a vacuum grease supported cover slip. Upon

observation under an optical microscope (Olympus BX51, 100X objective), a large number of micron-sized vesicles were observed.

Kinetics of the *de novo* phospholipid **3** synthesis

A 15 µL solution of lysolipid **2** (as 10 mM stock in 50 mM HEPES pH 7.5 buffer) was added to 120 µL of 50 mM HEPES pH 7.5 buffer. Afterward, a 15 µL solution of **1** (as 10 mM stock in 50 mM HEPES pH 7.5 buffer) was added and the mixture was stirred at 37 ^{o}C. Then, 20 µL aliquots of the reaction mixture were injected for HPLC analysis at various time points (0, 15, 30, 60 and 120 min). Progress of the reaction was monitored by measuring the integrated HPLC peak areas (205 nm) for lysolipid **2** and phospholipid **3**. The experiment was repeated in triplicate and a plot was made using the measured areas. The second order rate constants were obtained by monitoring the areas under 260 nm chromatogram corresponding to dodecanoyl-AMP peak.

Labeling of FadD10 with Alexa Fluor® 488

A glycerol stock solution of purified FadD10 (3.5 nmol) was initially exchanged in 100 mM sodium bicarbonate pH 8.3 using a 10 kDa MWCO microcentrifuge filter (Amicon Ultra, Merck Millipore). To 50 µL of the protein solution, 3.5 µL of a 1 mM stock solution of Alexa Fluor® 488 NHS Ester (3.5 nmol) (Thermo Fisher Scientific) in DMSO was added, and the resulting solution was stirred at rt for 1 h. The reaction mixture was washed 4 times with 100 mM HEPES pH 7.5 buffer to remove any unreacted dye and concentrated using a 10 kDa MWCO microcentrifuge filter. Finally, it was stored as a 10% glycerol stock solution where the concentration of the labeled protein was determined to be 14 µM from UV-Vis measurements (NanoDrop 2000C). It was verified by HPLC-ELSD-MS that the labeled enzyme is catalytically active.

Kinetics of phospholipid **3** formation assisted by FadD10

To a 20 µL solution of lysolipid **2** (10 mM stock in 50 mM HEPES pH 7.5 buffer) was successively added 90 µL HEPES (100 mM, pH 7.5), 18 µL of $MgCl_2$ (100 mM stock solution) and 6 µL of ATP (100 mM stock solution). Next, 4.4 µL of FadD10 (from a 10% glycerol stock, 6.75 mg/mL) were added, and the solution was mixed by gentle tapping. Afterward, 20 µL of dodecanoic acid (as 10 mM sodium dodecanoate stock solution) were added and the total volume was made up to 200 µL by adding requisite volume of water. The reaction mixture was kept incubated in a 37 ^{o}C water bath. Then, 20 µL aliquots were taken out at various time points (0, 1, 2, 3, 4, 5, 6 and 8 h) and 80 µL of MeOH were added. The solution was briefly centrifuged and the supernatant was collected and injected into HPLC. Progress of the reaction was monitored by measuring the integrated HPLC peak areas (205 nm) for lysolipid **2** and phospholipid **3**. The experiment was done in triplicate and a plot was generated from the measured areas.

Phospholipid **7** formation assisted by FadD10

A reaction was set up using 1.0 mM lysolipid **6**, 8.0 mM $MgCl_2$, 3.0 mM ATP, 1.0 mM sodium dodecanoate, and 10 µM FadD10 in 100 mM HEPES buffer (pH 8.0) at 37 ^{o}C.

Encapsulation of FadD10: evaluation using SDS-PAGE

Phospholipid **3** vesicles (1 mM lipid) were formed *in situ* according to the procedure described in the Methods section. Then, 20 µL of the reaction mixture was taken out for evaluating total protein content. The remaining vesicle solution (80 µL) was washed thoroughly with the reaction buffer using a 300 kDa MWCO spin filter (Nanosep, Pall Corporation) and concentrated to a volume of about 40 µL. The wash fractions were combined and concentrated using a 10 kDa MWCO spin filter to a volume of about 40 µL. The following were loaded onto a pre-cast 4-20% polyacrylamide gel (Mini-Protean, Bio-Rad): 20 µL reaction mixture (total enzyme), 8.0 µL vesicle fraction (encapsulated protein) and 8.0 µL wash fraction (unencapsulated enzyme). In each case, 7 µL of 4X loading dye (Laemmli Sample Buffer, Bio-Rad) were added to the sample and the total volume made up to 28 µL by adding requisite volume of water. The samples were heated to 95 ºC for 2 min, followed by cooling on ice and then brief centrifugation prior to loading onto gel. The gel was run for 30 min at 130 V and then stained with Coomassie Blue (Instant Blue®, CBS Scientific) for 1 h. The stained gel was washed with distilled water and imaged with a commercial scanner.

Membrane localization of FadD10 encapsulated in giant unilamellar vesicles (GUVs)

We adapted a previously published inverse emulsion method[5] for vesicle preparation. 10 µL of a 100 mM solution of 1,2-dioleoyl-*sn*-glycero-3-phosphocholine (DOPC) in $CHCl_3$ were added to a 1 mL glass vial, placed under N_2 and dried for 15 min to prepare a lipid film. Then, 1 mL of mineral oil was added and placed under N_2 to displace the air above the mineral oil. The resulting mixture was sonicated at rt for 1 h. Afterward, 100 µL of the phospholipid oil was added to a 1.7 mL Eppendorf tube. Then, 10 µL of the upper buffer [solution containing 0.5 mM ATP, 1.5 mM $MgCl_2$, 50 mM HEPES pH 7.5 buffer, 3.5% Ficoll 400 (w/v) and 0.7 µM labeled (Alexa Fluor® 488) FadD10] was added, and the resulting mixture was flicked and vortexed until it was a cloudy emulsion. The corresponding emulsion was added to a 1.7 mL Eppendorf tube containing 100 µL of the lower buffer [solution containing 0.5 mM ATP, 1.5 mM $MgCl_2$, 50 mM HEPES pH 7.5 buffer], so it floated on top. After waiting for 10 min, the sample was centrifuged for 10 min at 9000-10000 rcf. The sample was separated from the oil (either aspirating off the oil or using a syringe/needle to collect the sample from the bottom) and the GUVs encapsulating FadD10 were observed by fluorescence microscopy. The unencapsulated contents were removed by spin filtration.

The sample containing GUVs encapsulating FadD10 (~100 µL) was placed on a cell culture chamber slide (Lab Tek™ II, Thermo Fisher Scientific). A syringe pump (Harvard Apparatus) was used for slowly delivering a solution to the glass slide placed on a microscope. A 50 µL solution containing 0.1 mM of lysolipid **4**, 0.1 mM dodecanoic acid, 0.5 mM ATP, 1.5 mM $MgCl_2$ and 50 mM HEPES pH 7.5 buffer was loaded onto a syringe. The slide was placed on a microscope and the solution containing the lipid precursors was supplied at 15 µL/h. Initially, the green fluorescence from the labeled enzyme was observed throughout the volume of the vesicle. Gradually, it was observed that the fluorescence was mostly localized in the membrane (Supplementary Movie 2), thus indicating that FadD10 has preference for association with the membrane in the presence of the reactants. We made such observation with all (n = 49) GUVs under examination in three experiments. In a control experiment, where dodecanoic acid was

omitted, the labeled FadD10 leaked rapidly out of the vesicles (Supplementary Fig. 12d). In another control experiment, no precursors were added and no membrane association was observed (Supplementary Fig. 12e).

Phospholipid synthesis in vesicles encapsulating FadD10: HPLC determination

Vesicles composed of 2 mM phospholipid **3** and encapsulating FadD10 were prepared as described in the Methods section. Then, 2.5 µg Proteinase K were added to digest any unencapsulated enzyme. Into 50 µL of this vesicle solution, precursors were slowly added using a syringe pump (flow rate: 20 µL/h). The precursor solution (total volume: 50 µL) was composed of 1 mM lysolipid **4**, 1 mM dodecanoic acid, 3 mM ATP, 9 mM $MgCl_2$, 40 mM HEPES pH 7.5 buffer and 2.5% glycerol. The osmolarities were closely matched by osmometric measurements. The vesicles were kept incubated at 37 ºC. The vesicle solution was analyzed after 4-6 h by HPLC-ELSD-MS and compared with that before the start of the flow.

Supplementary References

1. Becerra, N., Toro, C., Zanocco, A. L., Lemp, E., Gunther, G. Characterization of micelles formed by sucrose 6-*O*-monoesters. *Coll. Surf. A*, **327**, 134-139 (2008).
2. Mullen, L. B. & Sutherland, J. D. Formation of potentially prebiotic amphiphiles by reaction of β-hydroxy-*n*-alkylamines with cyclotriphosphate. *Angew. Chem. Int. Ed.* **46,** 4166–4168 (2007).
3. Iancu, C.V. *et al.* Electron cryotomography sample preparation using the Vitrobot. *Nat. Protoc.* **1**, 2813-2819 (2006).
4. R. J. Brea, A. K. Rudd, N. K. Devaraj. Non-Enzymatic Biomimetic Remodeling of Phospholipids in Synthetic Liposomes. *Proc. Nat. Acad. Sci*, **113** (31), 8589-8594 (2016).
5. Pautot, S., Frisken, B. J. & Weitz, D. A. Engineering asymmetric vesicles. *Proc. Natl. Acad. Sci. U.S.A.* **100**, 10718-10721 (2003).
