# Supplementary material for: A minimal biochemical route towards de novo formation of synthetic phospholipid membranes
Source: Nat Commun. 2019 Jan 17;10:300. doi: 10.1038/s41467-018-08174-x (PMC6336818; doi:10.1038/s41467-018-08174-x)
Supplement: Supplementary file 3 — Description of Additional Supplementary Files [file 41467_2018_8174_MOESM3_ESM.docx]

**Description of Additional Supplementary Files**

**File Name**: Supplementary Movie 1

**Description**: In situ formation of vesicles by the reaction between dodecanoylAMP and lysolipid 2. An aqueous solution of 1 mM dodecanoyl-AMP (1) and lysolipid 2 was placed on a glass slide at 37 oC. Images were taken over a period of 30 min. Frames are 15 s apart. Video frame rate is 6 fps.

**File Name:** Supplementary Movie 2

**Description:** Time lapse fluorescence microscopy of membrane association of fluorescently labeled FadD10. An aqueous solution of unilamellar DOPC vesicles encapsulating fluorescently labeled (Alexa Fluor® 488) FadD10 was supplied with reactive precursors for phospholipid formation. Sequential fluorescence images were taken over a period of 30 min. Frames are 30 s apart. Video frame rate is 2 fps.

**File Name:** Supplementary Movie 3

**Description:** Time lapse fluorescence microscopy corresponding to vesicle growth in a microfluidic device. The vesicles were initially composed of phospholipid 3. Reactive precursors were supplied leading to the formation of phospholipid 5 and hence growth. Sequential fluorescence images (Texas Red® channel) were taken over a period of 12 h 10 min at 37 o C. The scale bar represents 10 µm. Frames are 5 min apart. Video frame rate is 7 fps. Fluorescence intensity is represented on a logarithmic scale to enhance visibility of internal membranous structures.

**File Name:** Supplementary Movie 4

**Description:** Time lapse fluorescence microscopy corresponding to vesicle growth trapped in a microfluidic device. The vesicles were initially composed of phospholipid 3. Reactive precursors were supplied leading to the formation of phospholipid 5 and hence growth. Sequential fluorescence images (Texas Red® channel) were taken over a period of 11 h 30 min at 37 o C. Frames are 5 min apart. Video frame rate is 7 fps. Fluorescence intensity is represented on a logarithmic scale to enhance visibility of internal membranous structures.

**File Name:** Supplementary Movie 5

**Description:** Time lapse fluorescence microscopy corresponding to vesicle division while maintaining internal content. Sequential fluorescence images were taken over a period of 10 h at 37 o C. Frames are 5 min apart. Video frame rate is 7 fps. Fluorescence intensity is represented on a logarithmic scale to enhance visibility of internal membranous structures.

**File Name:** Supplementary Movie 6

**Description:** Time lapse fluorescence microscopy corresponding to vesicle division while maintaining internal content. Sequential fluorescence images were taken over a period of 11 h at 37 oC. Frames are 5 min apart. Video frame rate is 7 fps. Fluorescence intensity is represented on a logarithmic scale to enhance visibility of internal membranous structures.

**File Name:** Supplementary Movie 7

**Description:** Time lapse fluorescence microscopy corresponding to the control experiment. Vesicles composed of phospholipid 3 were supplied with an unreactive lysolipid (Lyso C16:0 PC-OH) along with other precursors. Sequential fluorescence images (Texas Red® channel) were taken over a period of 8 h at 37 o C. Frames are 5 min apart. Video frame rate is 7 fps. Fluorescence intensity is represented on a logarithmic scale to enhance visibility of internal membranous structures.
